# Potential of FT-MIR/GC-MS Multivariate Hyphenation for the Fast Characterization of Agavins Metabolism

**DOI:** 10.1021/acs.analchem.5c01074

**Published:** 2025-07-16

**Authors:** Luis F. Salomé-Abarca, Ruth E. Márquez-López, Patricia A. Santiago-García, Mercedes G. López

**Affiliations:** † Colegio de Postgraduados-Campus Montecillo, Posgrado de Recursos Genéticos y Productividad-Fruticultura, Km 36.5 Carretera México – Texcoco, Montecillo, Texcoco de Mora 56230, Estado de México, Mexico; ‡ Instituto Politécnico Nacional, Centro Interdisciplinario de Investigación para el Desarrollo Integral Regional-Unidad Oaxaca, Oaxaca 71230, Mexico; § Departamento de Biotecnología y Bioquímica, Centro de Investigación y Estudios Avanzados del IPN-Irapuato, Km. 9.6 Libramiento Norte Carretera Irapuato-León, Irapuato 36824, Guanajuato, Mexico

## Abstract

Agavins are some of the most recently characterized fructans. FT-MIR is the fastest approach to detect them, and GC-MS provides valuable structural features. Thus, we explored the feasibility of integrating data from these two analytical platforms to characterize the metabolism of agavins from two emblematic agave species, *Agave angustifolia* and *Agave potatorum*. FT-MIR quickly provided information about the species and age variation. The analyses demonstrated for the first time the high efficiency of GC-MS glycosidic linkage analysis data for MVDA. Finally, specific MIR regions were correlated to structural features determined through GC-MS, which increased the level of structural information obtained by IR analyses. This approach could accelerate the study of agavins’ metabolism.

## Graphical abstract

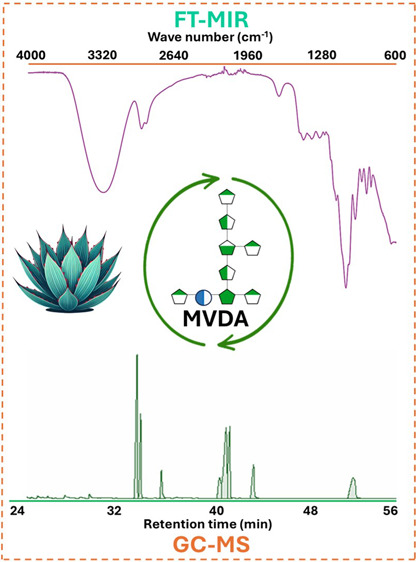

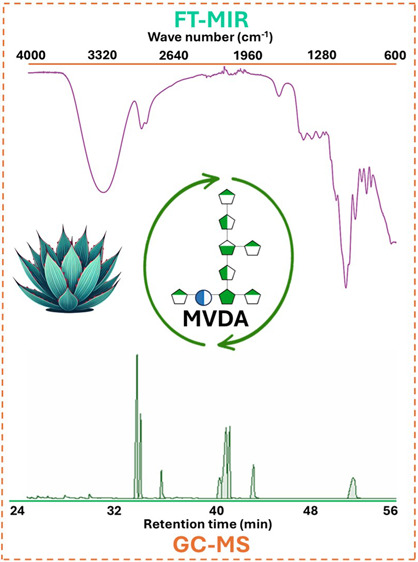

## Introduction

Fructans are polymers made of fructose, if any, with one glucose in their structures.^1^ Fructans are classified as linear inulins (β2→1) and levan (β2→6) with terminal glucose; neo-series of inulins and levan with internal glucose; moderate branched graminans (β2→1/β2→6); and agavins, highly branched neo-fructans.^2,3^ Agavins are of great interest because of their metabolic effects and correlation with tequila production.^4–6^ Thus, several studies have been conducted on the metabolism of agavins including their biosynthesis, accumulation, applications, and content in agave derived-products.^7–10^ To perform such characterizations, several analytical platforms have been employed, for instance, thin layer chromatography (TLC), high performance thin layer chromatography (HPTLC), high performance liquid chromatography (HPLC), matrix assisted laser desorption ionization mass spectrometry (MALDI-MS), Fourier transform mid infrared spectrometry (FT-MIR), gas chromatography coupled to mass spectrometry (GC-MS), high performance anion exchange chromatography coupled to pulsed amperometric detection (HPAEC-PAD), and nuclear magnetic resonance (NMR).^9–15^

Each of these techniques possesses advantages and drawbacks. Some examples include the parallel analysis of samples by TLC, but low reproducibility when manually performed. Even the implementation of HPTLC cannot properly separate agavins with a degree of polymerization (DP) higher than 12.^16^ HPLC provides total inulin content data, but it cannot separate agavins with DP higher than four.^13^ Conversely, HPAEC-PAD separates fructans with DP up to 40 units, but it does not provide specific structural data, is time-consuming, and needs standard compounds for identification and quantitation.^10^ Proton NMR (^1^H NMR) is limited to overlapped fructose signals and general DP information obtained by integrating all fructose signals in respect to the glucose anomeric proton.^2,9,15^ On the other hand, carbon and bidimensional experiments provide more valuable structural information, but they are time-consuming.^2,9,15^ GC-MS yields structural and molar contribution data; however, samples need complex and time-consuming derivatization, and the resulting structures depend on the predominant type of fructan in the extracts.^2,14^ Finally, FT-MIR possesses the fastest time of analysis, but its structural information is limited to functional groups.^17^

Each analytical platform offers a specific time and space view of these molecules. Nonetheless, the study of metabolites and biomolecules tends to become more efficient and more informative. This has been achieved by the creation of new technology or the on- and offline hyphenation of multiple analytical platforms.^16^ In this regard, FT-MIR gathers the fast analysis character of a high throughput approach, and GC-MS provides specific structural data based on glycosidic linkage analysis.^2^ Therefore, this research aimed to evaluate the efficiency of FT-MIR and GC-MS data for the characterization of agavin metabolism and assess the feasibility of their offline hyphenation through multivariate data analysis (MVDA). Finally, we characterize the effect of the type of agavins present in the extracts on the molecular structure prediction and MVDA output. To carry out such a task, we used a previously characterized sample set of fructans extracted from the emblematic *Agave potatorum* Zucc. and *Agave angustifolia* Haw. as the model.^18^

## Materials and Methods

### Plant Material

*Agave angustifolia* Haw. espadín from 1 to 6 years old were collected at San Esteban Amatlán, Oaxaca, Mexico (16°230 N and 96°300 W, 1500 m above mean sea level) (*n* = 18). Temperatures ranged from 8 to 28 °C in a semidry subhumid climate and the precipitation varied between 600- and 800 mm. Plant age was counted from the planting of the “hijuelo” (plant shoot) in the field. *Agave potatorum* Zucc. specimens from 1 to 6 years old were collected at Infiernillo, Zaachila Oaxaca, Mexico (16°890 N and 97°190 W, 1969 m above mean sea level) (*n* = 18). Temperatures ranged from 10 to 26 °C in a semiwarm subhumid climate and the precipitation varied between 300- and 1200 mm. Plant age was counted from the moment of seed sowing in the field. All specimens were collected during the rainy season, September–October 2021, free of water deficit.^18^

### Degrees Brix (°Bx)

Immediately after plant material collection, pine (“piña”) pieces were processed in a mill to obtain agave pine juice. Then, 300 μL of freshly extracted juice were placed on the measuring plate of a digital refractometer (HANNA, HI96801), which automatically provided the °Brix values.

### Fructan Extraction

This was performed as previously described^10^ with some modifications. Briefly, 100 g of plant materials was extracted with aqueous ethanol (80%) (1:1, v, m) at 60 °C under continuous stirring for 1 h (*n* = 36). The materials were re-extracted twice with 100 mL of distilled water for 30 min. The hydroalcoholic and water extracts were mixed, defatted with chloroform, and concentrated with a rotary evaporator (Büchi Rotovapor R-200, Brinkmann Instruments Inc., Westbury, NY). The complete fructan extracts (CF) were dried in a spray dryer device (Yamato, ADL 3115, Tokyo, Japan).^18^

### Fructan Fractionation

To get the agave fructooligosaccharides (aFOS) and high polymerization degree (HDP, DP > 12) fractions (*n* = 72), 500 mg of CFs was precipitated with cold absolute ethanol at 4 °C. To do so, CFs were resuspended in 5 mL of water, mixed with 25 mL of cold ethanol, and incubated at 4 °C overnight. After precipitation, the suspensions were centrifuged at 8000 rpm for 10 min. The upper phase and pellet contained aFOS and HDP-fructans, respectively. Both fractions were dried by lyophilization.

### Total Fructan Content (Tfru)

The Tfru, defined as the grams of fructan per 100 g of agave tissue, was enzymatically determined with the commercial “Fructans” (K-FRUC) test kit (Megazyme International Ireland, Ltd., Wicklow, Ireland) following the manufacturer instructions.^18^

### Fourier-Transformed Infrared (FT-IR) Spectroscopy

Infrared analysis was performed on a Nicolet 6700 FTIR spectrometer (Thermo Scientific) with a single bounce Smart ITR diamond ATR-accessory and a DTGS KBr detector. The IR measurements were recorded by placing 50 mg of each sample on an ATR crystal plate. For all samples (*n* = 108), 32 scans were performed between 4000 and 650 cm^–1^ with nominal resolution of 4 cm^–1^ in transmittance mode (%T). The MIR spectra were analyzed with OMNIC software V. 8.3.103 (Thermo Fisher Scientific Inc.). Data were used for spectra interpretation and MVDA. The spectral analysis lasted 4 h and MDVA around 2 days.

### Limit of Detection (LOD) and Limit of Quantification (LOQ)

These parameters were calculated as previously described by calculating the typical standard error xy (Sty) and slope (*m*) values and applying them in the formulas LOD = 3.3 (Sty/*m*) and LOQ = 10 (Sty/*m*).^19^ The Sty- and *m*-values were calculated from a calibration curve with linear range between 500 and 29.7 mg/mL made of commercial chicory FOS (Megazyme), *A. angustifolia,* and *A. potatorum* CFs. The *R* ^2^ coefficient for the FOS regression was 0.9986 with the slope equation *y* = 0.001*x* – 0.0082. The LOD and LOQ for FOS were 23.44 and 71.02 mg/mL, respectively. For *A. angustifolia*, the *R* ^2^ was 0.9989, equation *y* = 0.0016*x* – 0.0292, LOD = 19.83 mg/mL, and LOQ = 60.10 mg/mL. For *A. potatorum*, the *R* ^2^ was 0.9986, equation *y* = 0.0013*x* – 0.0222, LOD = 22.30 mg/mL, and LOQ = 67.60 mg/mL. As 4 μL of each solution were placed onto the ATR crystal, FOS, *A. angustifolia*, and *A. potatorum* LODs are equivalent to 93.76, 79.32, and 89.20 μg of each fructan source, respectively. LOQs of FOS, *A. angustifolia*, and *A. potatorum* correspond to 284.08, 240.40, and 270.40 μg of each fructan source, respectively, on the ATR crystal.

### Partially Methylated Alditol Acetates Derivatization (PMAAs)

Lyophilized CF extracts, aFOS, and HDP-fractions (10 mg, *n* = 54) were individually dissolved in 0.5 mL of dimethyl sulfoxide and stirred overnight for dissolution. Subsequently, their derivatization into PMAAs was performed as described for agave samples.^2,14^ The sample derivatization (nine samples per week) and GC analysis time lasted around 2 months, and the MVDA was around 3 days.

### Glycosidic Linkage Analysis by GC-MS

The analysis was performed in a gas chromatograph 7890B (Agilent Technologies) equipped with an automatic liquid sampler 7386B and a selective mass detector 5779A (Agilent Technologies). The separation occurred in an HP5-MS capillary column (30 m × 0.250 mm × 0.25 μm). All conditions were programed as previously described for analysis of fructan samples.^10^

### Multivariate Data Analysis

FT-MIR second derivative data were scrutinized by principal component analysis (PCA). Data were mean centered and scaled by unit variance (UV). The data set was also explored by orthogonal projection to latent structures discriminant analysis (OPLS-DA) setting agave species as classes and scaled by UV. Correlations between agave age, Tfru, °Bx, and the FT-MIR-fructan variation were established by orthogonal projection to latent structures (OPLS)-analysis scaled by UV. Only values obtained from the carbohydrate region (1300–900 cm^–1^) were used for the analyses. GC-MS data were analyzed by PCA scaled by UV. An OPLS-DA was performed to corroborate species differences. The similarity between types of fructan extracts was assessed by soft independent modeling of class analogy (SIMCA)-analysis. The CF extracts were set as an origin model to measure the distance to the model (DModX)-values (*D*_crit_ ≤ 0.05).^11^ Correlations between agave age, Tfru, and °Bx with the variation in glycosidic linkage patterns were determined by OPLS scaled by UV. Data for all GC-MS analyses consisted of PMAA’s peak areas normalized to total area. For FT-MIR/GC-MS data correlations, each PMAA chromatographic peak area was used as quantitative “Y” data and the FT-MIR raw (nonderived) data was set as “X” variables in OPLS models using CF extracts, aFOS, and HDP-fructan fractions. All OPLS-DA and OPLS models were statistically validated through permutation (100 permutations, *Q* ^2^ ≥ 0.40) and CV-ANOVA (*p* < 0.05) tests. Correlated variables were determined with a predictive variable importance for the projection (VIP-pred)-plot (VIP-pred ≥ 1).^11^

## Results and Discussion

The FT-MIR spectra of *Agave angustifolia* and *Agave potatorum* showed the same MIR-bands. The region between 4000 and 2500 cm^–1^ showed a broad band around 3300 cm^–1^ attributed to high abundance of hydroxyl groups in polysaccharides.^18^ Nevertheless, this band could also be indicative of O–H stretching vibrations in polyphenols, saponins, or free sugars also present in agaves.^20^ Two bands between 2900 and 2800 cm^–1^ corresponded to asymmetric stretching vibrations of CH_2_ and stretching vibrations of skeletal CH in polysaccharides.^21^ The double bonds stretching region (1800–1500 cm^–1^) did not show the typical C=O stretch above 1700 cm^–1^, attributed to carbonyl groups, which suggested that carbohydrates occur exclusively in their cyclic forms^22^ (Figure S1).

The local symmetry region (1500–1200 cm^–1^) showed deformational vibrations of CH_2_, C–O–H deformations, and C–C–C stretching commonly found in carbohydrates.^23,24^ Overlapped bands around 1600 cm^–1^ have been reported as potential CO-NH groups in barley fructan extracts.^25^ The fingerprinting region (1200–800 cm^–1^) showed several strong bands produced by C–O stretching, C–O–H, and C–O–C bending.^24^ Bands between 1175 and 1140 cm^–1^, not present in monosaccharides, have been associated with glycosidic linkages in polysaccharides.^26^ Thus, depending on the polysaccharide structure, different bands’ arrangements might be observed.^27^ In this case, a band around 1140 cm^–1^ and another band around 1020 cm^–1^ flanked by three bands at 1120, 985, and 925 cm^–1^ corresponded to the typical MIR-pattern of fructans.^18,28,29^ The bands at 1120 and 1020 cm^–1^ have been associated with stretching vibrations of C–O–C groups in furanosyl residues.^30,31^ Furthermore, the band at 925 cm^–1^ has been related to β(2→1) moieties in inulin chains.^28,29^ Finally, weak bands at 870, 830, and 755 cm^–1^ were assigned to combined vibrations of C–C, C–O, and C–H bonds in free fructose^32^ (Figure S1).

The IR data were further scrutinized by PCA. The score plots of aFOS, HDP-fructans, and CF showed good separation between species along the PC1. The best separation according to species factors was achieved by models constructed from CF and HDP data (Figure S2A,B). Both data sets explained around 90% of the total variation of the model. The CF and HDP models produced 5 PCs, and PC1 in CF and HDP captured 58 and 66% of the total variation, respectively. The models also separated samples according to age between PC2 and PC3 (Figure S2C,D). Interestingly, only CF and HDP models showed a tighter cluster in *A. angustifolia* samples indicating that HDP-fructans, in this species, possess less infraspecific variation than those in *A. potatorum*. This indicated that species-specific differences are determined by HDP-agavins. That is, agave species are mainly differentiated because of their DP-range.

To gain deeper insights into species factors, the data sets were analyzed by OPLS-DA. In this model, agave species were treated as distinct classes, and the analysis was conducted on individual data sets. The CF data produced the best model for differentiating agave species (*Q* ^2^ = 0.85 and *p* = 1.48 × 10^–10^) (Figure S3A). The S-plot of the model indicated that *A. angustifolia* produced higher transmission between 930 and 916 cm^–1^, an indicative of higher fructan content^28,29^ (Figure S3B). In this regard, it has been reported that *A. angustifolia* produces around 100 mg more fructans per gram of tissue than *A. potatorum*.^18^

To eliminate species and extract effects, the data sets were individually analyzed per species and extracts. The PCA analyses showed that aFOS models produced the best specimen classification according to their age (Figure S2E,F). Thus, age effects modulate the type and accumulation of aFOS. The effects of species and age factors on agavin variation have been documented for these agave species, which corroborated the wellness of the acquired data and modeling.^11,18^

Continuing with the characterization of aging affects, an OPLS model was constructed using agave age as the quantitative “Y” variable. Data sets from CF, HDP, and aFOS were individually analyzed for *A. potatorum* and *A. angustifolia*. *Agave potatorum* showed the best age correlation models using data from CF extracts (Figure S4A), while *A. angustifolia* obtained the best age correlation with aFOS fractions (Figure S4B). Models for *A. potatorum* and *A. angustifolia* obtained *Q* ^2^ values of 0.77 and 0.87, and *p*-values of 0.004 and 0.01, respectively. This indicated that aFOS variation and accumulation occurred more linearly in *A. angustifolia*. This was also observed by comparing the total fructan (Tfru) values produced by each species over 6 years. For instance, every year, *A. angustifolia* gains around 100 mg of Tfru per gram of tissue, while Tfru accumulation in *A. potatorum* was more heterogeneous, especially in its first 3 years.^18^ This corroborated that aFOS correlates better with age increase, which was in line with PCA analyses.

In this context, Tfru variation through age could be parallelly reflected in the variation of brix degrees (°Bx). For instance, even if there were no significant differences in the increase of °Bx through age in *A.* spp., *A. salmiana* var. chino, *A. tequilana* var. cenizo, and *A. salmiana* spp. crassipina, there was a clear trend in the increase of their °Brix.^33^ The lack of significant differences in that study might be related to the mixing of samples of three ages. In this case, samples were individually processed by age, which could potentiate differences among the ages. Thus, OPLS models were built for scrutinizing the correlation among Tfru, °Brix, and MIR-carbohydrate data variation through time. The analyses showed a good correlation between the carbohydrate data and both variables. For *A. potatorum* (Figure S4C) and *A. angustifolia* (Figure S4D), the obtained *Q* ^2^ values for the Tfru models were 0.81 and 0.82, respectively, and *p*-values <0.001. For °Bx models, the *Q* ^2^ and *p*-values were 0.79 and 0.83, and 0.0001 and 0.002, respectively for *A. potatorum* (Figure S4E) and *A. angustifolia* (Figure S4F). Interestingly, the VIP-pred-plot determined that the age, Tfru, and °Brix models shared several MIR regions as variables correlated to each of them. For instance, the ranges 934–935, 990–996, 1008–1010, 1057–1064, 1076–1080, 1203–1206, 1220–1222, and 1235–1238 cm^–1^ in *A. angustifolia* (Tables S1–S3). Nonetheless, the °Bx model showed a distinctive region between 1143 and 1144 cm^–1^ assigned to an asymmetric stretch of a methylene ether bond (−C–O–C−) associated with glycosidic linkages in polysaccharides.^34^ Considering that °Bx is an easy and accessible measurement, it would be worth exploring as an indirect way of measuring total fructan contents.

Something similar occurred with *A. potatorum* OPLS models, which also shared several correlated MIR regions. These included 953–957, 975–980, 997–999, 1090–1095, 1127–1131, and 1172–1178 cm^–1^. However, for this species, each model showed distinctive ranges. The age model showed the range 1023–1026 cm^–1^, while the Tfru model showed the 946–947 cm^–1^ range. The °Bx model provided three distinctive ranges, 1010–1012, 1060–1061, and 1217–1218 cm^–1^ (Tables S4–S6). Bands between 1200 and 900 cm^–1^ have been related with C–O–C vibrations in cyclic structures suggesting high-carbohydrate content.^25^ In addition, all analyses showed that a metabolic differentiation occurred over 3 years. This was observed as a break in the continuity of all OPLS models between 3 and 4-year-old specimens (Figure S4). This phenomenon has been documented in the aFOS fraction of both species analyzed by HPTLC.^11^

Thus, far, FT-MIR is a practical and fast fingerprinting approach for agave metabolism and its correlation to important parameters to produce distillated beverages.^5^ Nonetheless, to deeply understand these metabolic changes, more structural data are needed. In this regard, GC-MS has been proven as a valuable technique for getting structural data of fructans through partially methylated alditol acetate (PMAA)-derivatives.^2,10,14,35^

Considering the correlation between agaves’ carbohydrate variation and age, and their metabolic differentiation at 3 years, aFOS, HDP, and CF extracts from 1, 3, and 6 year old specimens were derivatized to PMAAs and analyzed by GC-MS. Both agave species showed the typical chromatographic PMAA profile of agavins.^2,10,12^ That is, in all sample types, there were eight peaks corresponding from left to right to epimers of terminal fructose, terminal glucose, inulin, levan, internal glucose, and branching fructose (Figure S5). Mass data of the glycosidic linkage analysis are compiled in Table S7. Structures produced from these peaks showed differences depending on the type of fructan extract and sample age in both agave species. The HDP derivatives showed the most differentiated trend including elongation of the inulin chain, increase of branching points, and apparition of levan ramifications through age (Figures S6 and S7). Such a trend has been reported in *A. tequilana* specimens.^10^

However, the chemical structures that resulted from aFOS-PMAAs were the most similar to those obtained from CF extracts. According to the concept of structure prediction based on PMAAs,^2^ this result indicates that CF extracts possess a higher number of aFOS molecules than HDP molecules. That is, predicted structures from PMAAs represent an average of all molecules in the extract; thus, a sample with a higher proportion of aFOS will predict molecules with structural patterns like aFOS. As these molecules are short, their DP and isomer variation is limited and more difficult to differentiate when dealing with general predicted structures. Conversely, PMAAs obtained from HDP fractions provided more differentiated agavin structures through aging in both agave species. This might be associated with their larger DP-range and isomer forms, which increase as the agave age increases too.^2,10^ Therefore, HDP fractions provided more information than aFOS regarding agavin structural variations through age.

Thus, far, PMAAs have been used only for determining and describing general fructan structures,^2,10,12^ but their chromatographic data have never been used for MVDA. Therefore, PAAMs data from extract types were scrutinized by this approach. The PCA of aFOS from *A. potatorum* and *A. angustifolia* showed the best separation by species (Figure 1A,B). The model produced 4 PCs explaining 83% of the total variation of the model (*RX* ^2^cum = 0.83) and it was validated by OPLS-DA modeling (*Q* ^2^ = 0.92 and *p* < 0.001). Moreover, the data set was individually analyzed by species showing that PMAAs were able to distinguish between aFOS, HPD, CF, and specimens’ age (Figure 1C,D). The PCA model for *A. potatorum* produced 3 PCs explaining 91% of the total variation of the model (*RX* ^2^cum = 0.91). The PCA for *A. angustifolia* also produced 3 PCs but explained only 83% of the total variation (*RX* ^2^cum = 0.83). Additionally, their DModX distances, obtained by SIMCA, were shorter than those of HDP fractions corroborating that aFOS are more similar to CF extracts (Figure 1E,F). This was in line with the structural prediction data.

**Figure 1:**
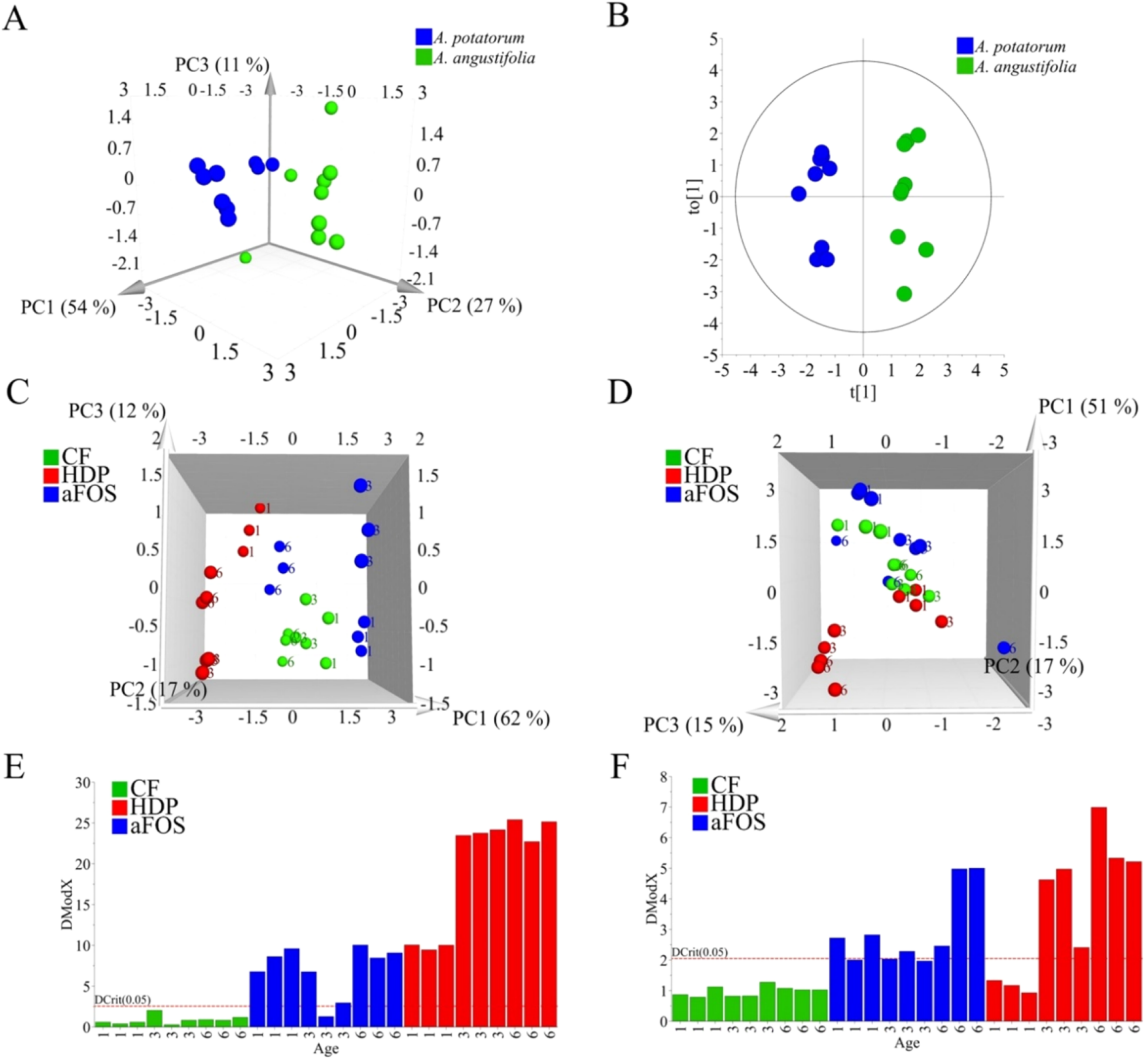
Multivariate data analysis of PMAAs obtained from complete fructan extracts (CF), agavin fructooligosaccharides (aFOS), and high polymerization degree (HDP)-fructans from *Agave potatorum* and *Agave angustifolia*. (A) PCA analysis of aFOS from 1, 3, and 6-year-old specimens of *A. potatorum* and *A. angustifolia*. (B) OPLS-DA analysis of aFOS from 1, 3, and 6-year-old specimens of *A. potatorum* and *A. angustifolia*. (C) PCA of CF extracts, aFOS and HDP-fructans from 1, 3, and 6-year-old specimens of *A. potatorum*. Numbers indicate the agave age. (D) PCA of CF extracts, aFOS and HDP-fructans of *A. angustifolia*. (E) Soft independent modeling of class analogy (SIMCA)-analysis of CF extracts, aFOS, and HDP-fructans of *A. potatorum*. (F) SIMCA analysis of CF extracts, aFOS, and HDP-fructans of *A. angustifolia*.

The GC-MS data were further scrutinized by OPLS modeling for the correlation between structural variation, age, Tfru, and °Bx. The best models for age correlation were produced by aFOS in both agave species (Figure S8A,B). The models’ *Q* ^2^ values were 0.97 and 0.98, and *p*-values of 0.02 and 0.001, respectively for *A. potatorum* and *A. angustifolia*. For Tfru correlations, the best models were also produced by aFOS (Figure S8C,D) with *Q* ^2^ values of 0.97 and 0.99, and *p*-values of 0.02 and 0.0006, respectively for *A. potatorum* and *A. angustifolia*. Among the °Bx models, the CF data produced the best one for *A. potatorum* (*Q* ^2^ = 0.96, *p* = 0.00004) (Figure S8E). For *A. angustifolia*, the best model was produced by aFOS (*Q* ^2^ = 0.91, *p* = 0.02) (Figure S8F).

Additionally, the VIP-pred-plot determined that the length of inulin chain, branching degree, number of terminal fructose, and levan ramifications are the most correlated structural features of agavins with age increase in *A. potatorum* (Figure S9A). This represented an increase in the agavin DP and their complexity. In the case of *A. angustifolia*, all previous features, except for inulin chain length, were the most correlated structural features with age. Furthermore, *A. angustifolia* showed a correlation between age and the content of terminal glucose (Figure S9B). This indicated that agavins in this species could possess shorter inulin chains than those of *A. potatorum* but higher branching frequency and longer levan branches along with a higher activity of sucrose:fructan 6-fructosyltransferase (6-SFT; EC 2.4.1.10). The correlation between terminal glucose and age indicated an important variation in the graminan:agavin ratio, favoring agavins, as the age of *A. angustifolia* increases. This implied an increase in the activity of fructan:fructan 6G-fructosyltransferase (6G-FFT; EC 2.4.1.243), responsible for synthetizing neo-series.^36^ Differently, larger inulin chains in *A. potatorum* suggested a higher activity of fructan:fructan 1-fructosyltransferase (1-FFT). This demonstrated the power of GC-MS analysis to discriminate metabolic features between agave species. Therefore, the structural classification obtained by GC-MS can serve as an alternative tool to assign different agave materials for specific industrial purposes, for instance, aFOS-rich agaves for prebiotic production and high-DP-rich agaves as food additives or encapsulant agents.^33^

As expected, the same variables were correlated in the same magnitude for the correlation of agavin structural variation through age and Tfru (Figure S9C,D). Contrastingly, the VIP-pred-plot determined a different correlation order in the °Brix OPLS models. In the case of *A. potatorum*, internal and terminal glucose were the only two features not correlated to °Bx (Figure S9E). This meant that carbohydrates other than graminans or agavins dictate the °Bx variation in *A. potatorum* through age. In *A. angustifolia*, levan moieties and internal glucose were not correlated with the increase of °Brix through agave age (Figure S9F). This suggested that carbohydrates determining °Brix differences through age possess similar levan branch lengths, and the ratio between graminans and agavins might affect the increase of °Brix in this species. Thus, considering FT-MIR and GC-MS data, we can state that °Bx might be a good Tfru content predictor, however, with limited potential to differentiate fructan structural variation.

For the first time, PMAAs data have been demonstrated as a tool not only for generating fructan structures but as a valuable chemometric data set. Nonetheless, this approach is time-consuming and needs specialized technical training. Therefore, establishing correlations between GC-MS and FT-MIR data could provide more structural meaning to specific wave numbers or regions, which could be later used for IR data interpretation. To assess the potential correlation between FT-MIR and GC-MS data, OPLS models were constructed setting GC-MS data (peak areas) as “Y” variables, and FT-MIR data (wave numbers) as “X” variables. For *A. potatorum*, only aFOS data produced validated results. The models for correlating inulin chains, levan chains, and branching degree produced *Q* ^2^ = 0.98, 0.96, and 0.95, respectively and *p* ≤ 0.01 (Figure 2A–C). Interestingly, most of the MIR ranges correlated with those features were overlapped. The ranges included 949–950, 954–956, 995, 1024–1028, 1037–1039, 1077–1078, 1091–1095, and 1106–1107 cm^–1^ (Tables S8–S10). Bands around 950 cm^–1^ have been pointed as characteristic α- and β-anomers signals of cyclic carbohydrates.^22^ Transmission at 995 cm^–1^ was attributed to coupled vibrations of a −O–C–O–C–O– system. The overlapping ranges might be explained by intrinsic correlations between those structural features. For instance, increasing of the branching points indicates a parallel increase of the inulin chain length. In addition, there will be new levan moieties at each branching point.^2,10^ However, an increase in levan moieties must be interpreted, along with the number of branching points. That is, an equal increase in both features might indicate more branches with short levan chains. Conversely, a small number of branching points and a high amount of levan moieties suggest less ramifications with larger levan chains.

**Figure 2:**
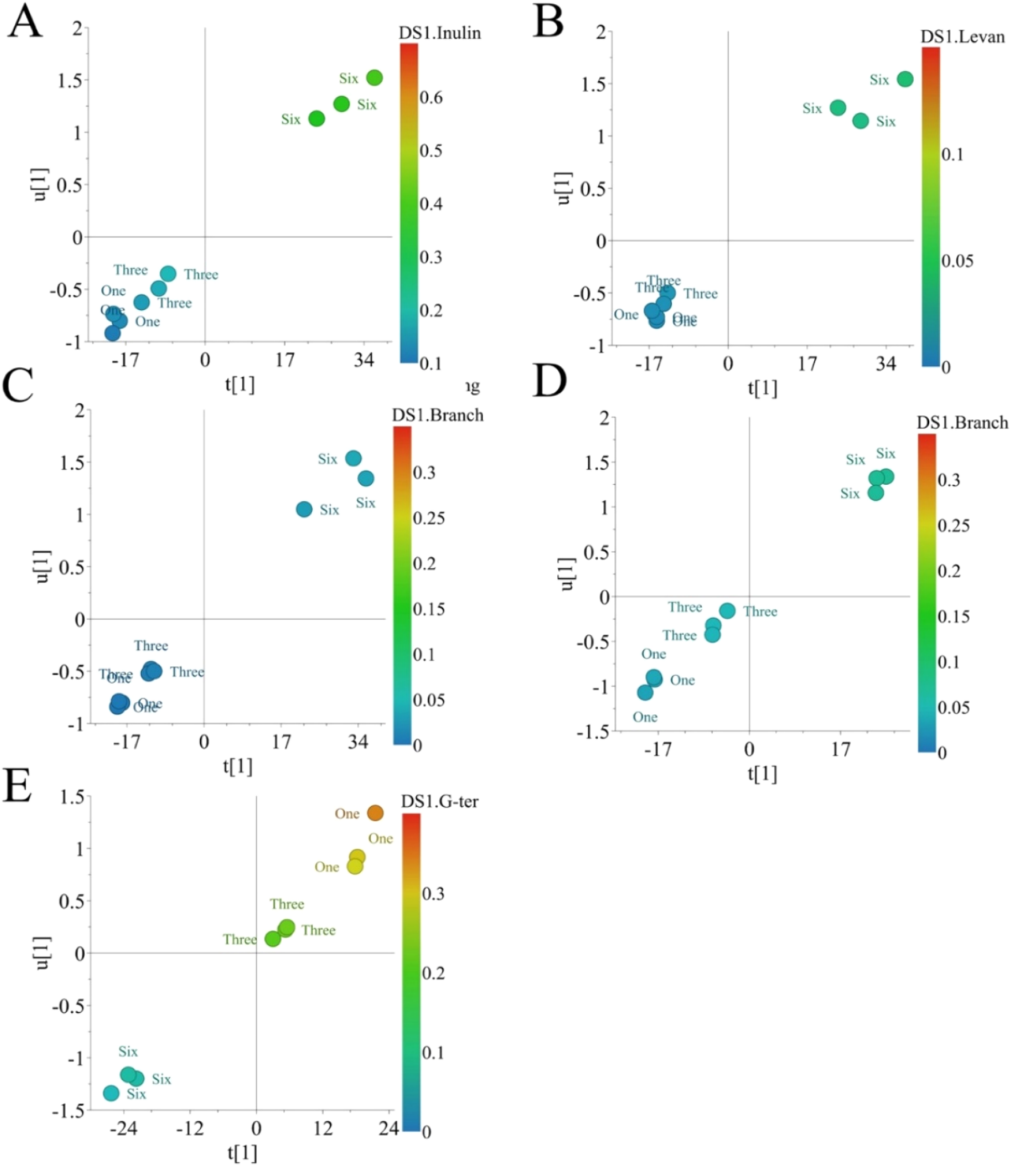
Correlation analysis between FT-MIR and GC-MS data through OPLS analysis. (A) OPLS analysis for the correlation between inulin moieties and FT-MIR data from aFOS of *Agave potatorum*. (B) OPLS analysis for the correlation between levan moieties and FT-MIR data from the aFOS of *A. potatorum*. (C) OPLS analysis for the correlation between the branching degree and FT-MIR data from aFOS of *A. potatorum*. (D) OPLS analysis for the correlation between branching degree and FT-MIR data from aFOS of *Agave angustifolia*. (E) OPLS analysis for the correlation between terminal glucose and FT-MIR data from aFOS of *A. angustifolia*.

There were distinctive MIR regions correlated to each GC-MS structural feature *in* *A. potatorum*, for instance, the range 919–920 cm^–1^ for the increase of inulin chain through age. As previously mentioned, this band has been correlated to the increase of inulin β(2→1) moieties.^25^ The range 978–1022 cm^–1^ was specific for changes in levan linkages through age. Finally, the range 1169–1170 cm^–1^ was correlated to branching degree variation through age. These bands corresponded to valent stretching vibrations of C–O–C groups and ring vibrational modes in cyclic structures.^37,38^

For *A. angustifolia*, there were only two validated models, branching degree and terminal glucose, obtained from aFOS data (Figure 2D,E). The branching (*Q* ^2^ = 0.98, *p* = 0.006) and terminal glucose (*Q* ^2^ = 0.96, *p* = 0.02) models were well validated. According to the VIP-pred plot, the top 50 wave numbers associated with aFOS branching variation through age included 1008–1007, 1039–1038, 1047–1043, 1060–1058, 1067–1064, 1075–1074, 1143–1142, 1206–1205, 1224–1222, 1234–1233, and 1238–1237 cm^–1^ (Tables S10 and S11). The range 1047–1038 cm^–1^ can be assigned to a C–O stretch of an acetal group located at the anomeric carbon of a sugar unit.^34^ The ranges 1075–1074 and 1143–1142 cm^–1^ were assigned to a C–O stretch of a primary OH bonded to a carbon and a C–O stretch in a cyclic/aliphatic ether linkage, respectively. The top ten correlated wave numbers to branching degree were 1237–1238, 1065–1066, 1044–1045, and 1038 cm^–1^. The ten most correlated wave numbers for terminal glucose were the same as for the branching degree. This suggested that both ranges are correlated to conserved structural features related to the age increase. Additionally, considering the top 50 correlated signals, there were distinctive ranges for terminal glucose (935, 991–992, and 1169–1167 cm^–1^). Bands between 990 and 970 cm^–1^ are produced by coupled vibrations in a −O–C–O–C–O– system, including an ether group in the furanosyl cycle, its anomeric carbon, and the aliphatic ether bond between fructose and glucose.^22^ A band at 1170 cm^–1^ has been related to C–O–C vibrations also in cyclic structures.^37^ It is worth noting that the OPLS model for terminal glucose showed an inverse age correlation. That is, terminal glucose decreased as the age increased, indicating that agavins become the predominant fructans in *A. angustifolia* as it gets older. Such a trend has been documented in *A. tequilana*.^10^ These data confirm and expand the results found by GC-MS standing alone.

The validated correlations in *A. potatorum* and *A. angustifolia* were in line with the molar contribution variation of each moiety in the fructan structure. For instance, a clear increase in the molar contributions of β(2→1), β(2→6), and branched fructose was observed as the age of this species increased (Figure S5A). Conversely, other moieties in the HDP and CF extracts showed no linear increase. Moreover, the molar contribution of terminal glucose and branched fructose in aFOS extracts of *A. angustifolia* increased as the agave age increased too (Figure S4). Despite the linear increase of most moieties, there was no increase in terminal fructose. This suggested that fructans might experience branch elongation rather than new branch formation. This has been reported for *A. fourcroydes*, *A. cantala*, and *A. tequilana*.^2^

## Conclusions

FT-MIR and GC-MS are robust fingerprinting platforms for the characterization of the agave fructan metabolism. FT-MIR determined that agave species are mainly differentiated by their DP-range, especially by HDP-agavins. The lower infraspecific variability of HDP-fructans conferred on them good species-biomarker character. Differently, aFOS represents a better age differentiator in agaves. GC-MS approached by MDVA provided specific linkage variation correlated to age increase, Tfru, °Bx, agave species, and age. Such correlations dilucidated an activity increase of 6G-FFT and 6-SFT in *A. angustifolia*, and 1-FFT in *A. potatorum*. Furthermore, correlations established between FT-MIR and GC-MS data corroborated the value of combining these analytical platforms by providing a more structural meaning to MIR signals. This positioned FT-MIR a step further from a common fingerprinting tool. Nonetheless, studies including a larger number of samples must be carried out to produce more robust models. FT-MIR/GC-MS correlations are species-dependent; thus, such correlations must be created for individual agave species. Finally, these correlations proved that most of the agavin structural variation in agave species through age can be profiled by aFOS. This highlights aFOS as the most informative block for the fast fingerprinting of agavins’ metabolism.

## Supplementary Material



## Notes

FT-MIR spectra and their MVDA. GC-MS PMAA-chromatograms, supervised MVDA, and molar contribution graphs. Tables with PMAA-MS data and tables with detailed correlated variables for each model. (PDF)
