# Supplementary material for: Potential of FT-MIR/GC-MS Multivariate Hyphenation for the Fast Characterization of Agavins Metabolism
Source: Anal Chem. 2025 Jul 16;97(33):17954–61. doi: 10.1021/acs.analchem.5c01074 (PMC12392261; doi:10.1021/acs.analchem.5c01074)
Supplement: Supplementary file 1 [file ac5c01074_si_001.pdf]

Supporting materials

The potential of FT-MIR/GC-MS multivariate hyphenation for the fast characterization of agavins metabolism

Luis F. Salomé-Abarca [1,§], Ruth E. Márquez-López [2,§], Patricia A. Santiago-García [2], Mercedes G. López [3,*]

[1]Colegio de Postgraduados-Campus Montecillo, Posgrado de Recursos Genéticos y Productividad-Fruticultura. Km 36.5 Carretera México - Texcoco, Montecillo, Texcoco de Mora 56230, Estado de México, México.

[2]Instituto Politécnico Nacional, Centro Interdisciplinario de Investigación para el Desarrollo Integral Regional-Unidad Oaxaca, Oaxaca 71230, México.

[3]Centro de Investigación y Estudios Avanzados del IPN-Irapuato, Departamento de Biotecnología y Bioquímica, Km. 9.6 Libramiento Norte Carretera Irapuato-León, Irapuato 36824, Guanajuato, México.

Table of contents

**Supplementary figure S1**	Typical FT-MIR spectra of CF, aFOS, and HDP-fructans of *Agave potatorum* and *Agave angustifolia*.
**Supplementary figure S2**	Multivariate data analysis of FT-MIR spectra of fructan extracted from *Agave potatorum* and *Agave angustifolia*.
**Supplementary figure S3**	Supervised multivariate data analysis of fructans of *Agave potatorum* and *Agave angustifolia*.
**Supplementary figure S4**	Correlation multivariate data analysis of fructans extracted from *Agave potatorum* and *Agave angustifolia*.
**Supplementary figure S5**	Typical chromatogram obtained after partially alditol acetate derivatization of agavins.
**Supplementary figure S6**	Predicted general agavin structures from PMAA-derivates of CF, aFOS, and HDP-fructans of one, three, and six-year-old specimens of *Agave angustifolia*.
**Supplementary figure S7**	Predicted general agavin structures from PMAA-derivates of CF, aFOS, and HDP-fructans of one, three, and six-year-old specimens of *Agave potatorum*.
**Supplementary figure S8**	Correlation multivariate data analysis of fructans from *Agave potatorum* and *Agave angustifolia* derivatized to PMAAs.
**Supplementary figure S9**	VIP*pred*-plot of OPLS analysis for the correlation between PMAA-derivatives data and agave age, Tfru, and ºBrix.

**Supplementary figure S10**	Molar contribution for each glycosidic linkage type present in the PMAA-derivates chromatogram of *Agave potatorum* and *Agave angustifolia*.
**Supplementary table S1**	Mass fragmentation data of glycosidic linkages present in *Agave potatorum* and *Agave angustifolia*.
**Supplementary table S2**	Top 50 correlated variables of the OPLS model for correlating agave age and FT-MIR data of *Agave angustifolia*.
**Supplementary table S3**	Top 50 correlated variables of the OPLS model for correlating agave Tfru and FT-MIR data of *Agave angustifolia*.
**Supplementary table S4**	Top 50 correlated variables of the OPLS model for correlating agave ºBrix and FT-MIR data of *Agave angustifolia*.
**Supplementary table S5**	Top 50 correlated variables of the OPLS model for correlating agave age and FT-MIR data of *Agave potatorum*.
**Supplementary table S6**	Top 50 correlated variables of the OPLS model for correlating agave Tfru and FT-MIR data of *Agave potatorum*.
**Supplementary table S7**	Top 50 correlated variables of the OPLS model for correlating agave ºBrix and FT-MIR data of *Agave potatorum*.
**Supplementary table S8**	Top 50 correlated variables of the OPLS model for correlating GC-MS inulin moieties and FT-MIR data of *Agave potatorum*.
**Supplementary table S9**	Top 50 correlated variables of the OPLS model for correlating GC-MS levan moieties and FT-MIR data of *Agave potatorum*.
**Supplementary table S10**	Top 50 correlated variables of the OPLS model for correlating GC-MS branching moieties and FT-MIR data of *Agave potatorum*.
**Supplementary table S11**	Top 50 correlated variables of the OPLS model for correlating GC-MS terminal glucose linkages and FT-MIR data of *Agave angustifolia*.
**Supplementary table S12**	Top 50 correlated variables of the OPLS model for correlating GC-MS branching moieties and FT-MIR data of *Agave angustifolia*.

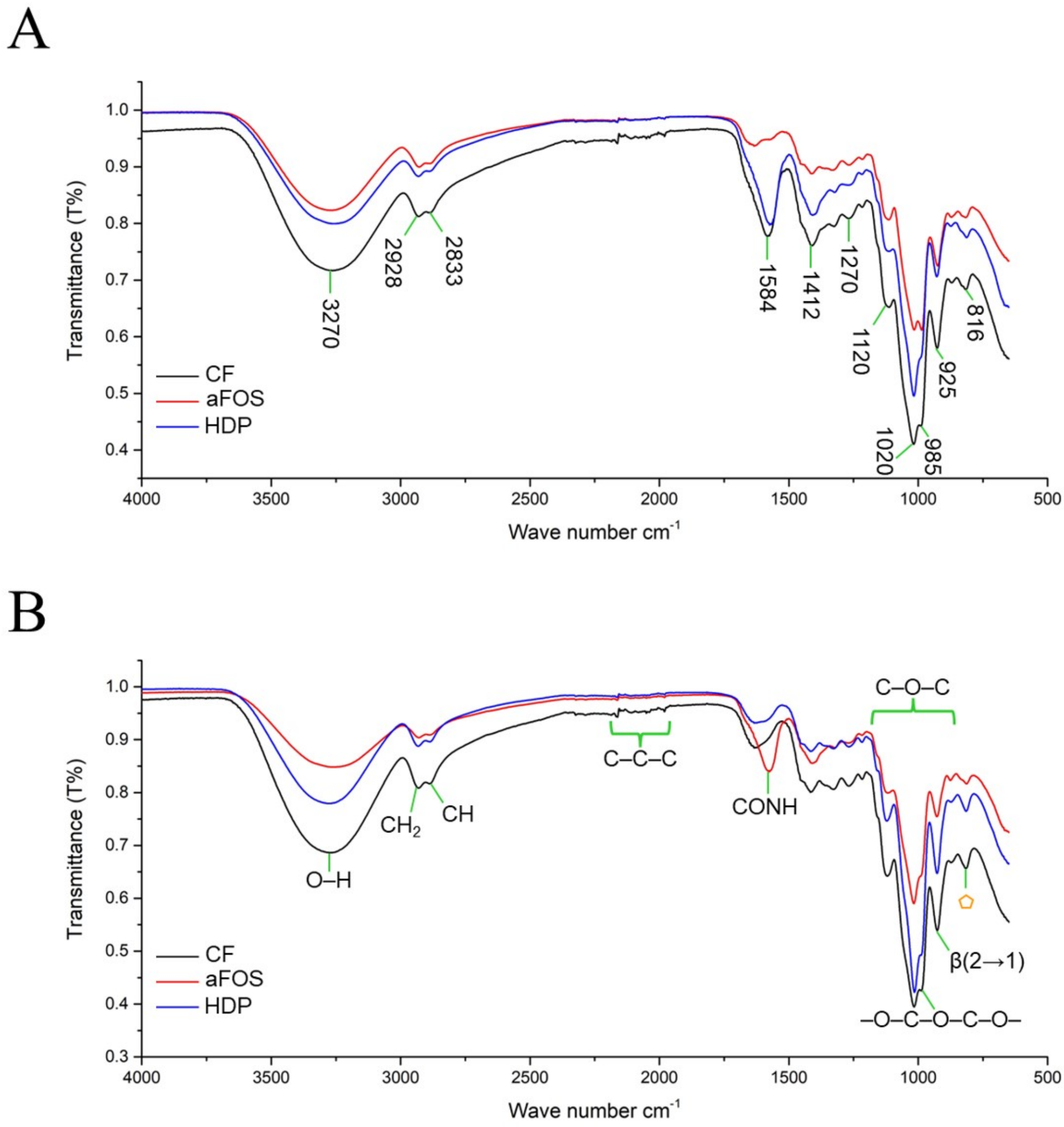

Supplementary figure S1. Typical FT-MIR spectra of complete fructan extracts (CF), aFOS, and HDP-fructans of **(A)** *Agave potatorum* and **(B)** *Agave angustifolia*. *Agave potatorum* spectra show wave numbers for each outstanding IR band, and *A. angustifolia* spectra shows functional groups assigned to each band. A pentagon represents free fructose.

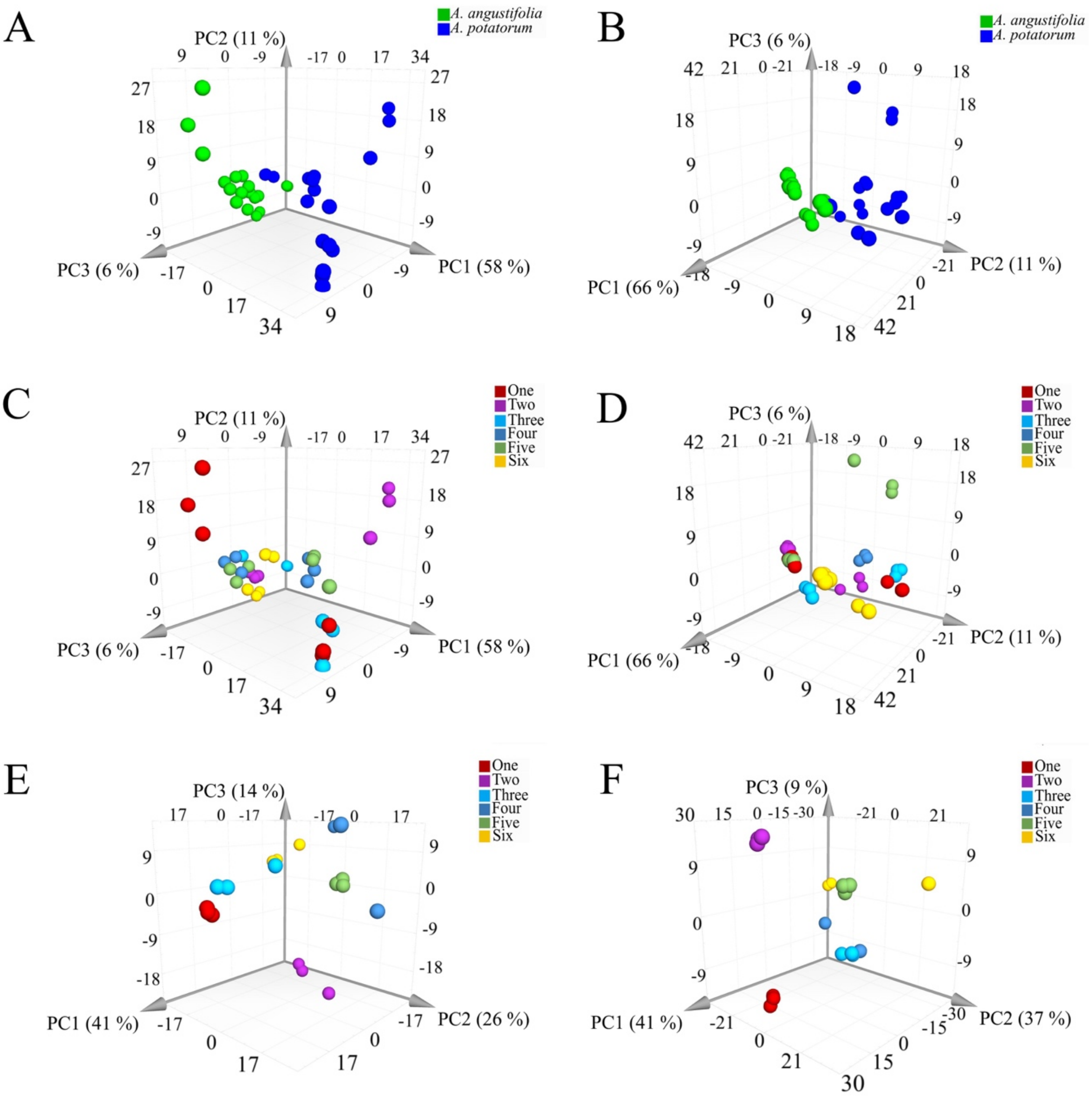

Supplementary figure S2. Multivariate data analysis of FT-MIR spectra of fructan extracted from *Agave potatorum* and *Agave angustifolia*. **(A)** Principal component analysis (PCA) of CF colored according to agave species. **(B)** PCA of high polymerization HDP-fructans colored according to agave species. **(C)** PCA of CF extracts colored according to agave age. **(D)** PCA of HDP-fructans colored according to specimens' age. **(E)** PCA of *A. potatorum* aFOS colored according to specimens' age. **(F)** PCA of *A. angustifolia* aFOS colored according to specimens' age.

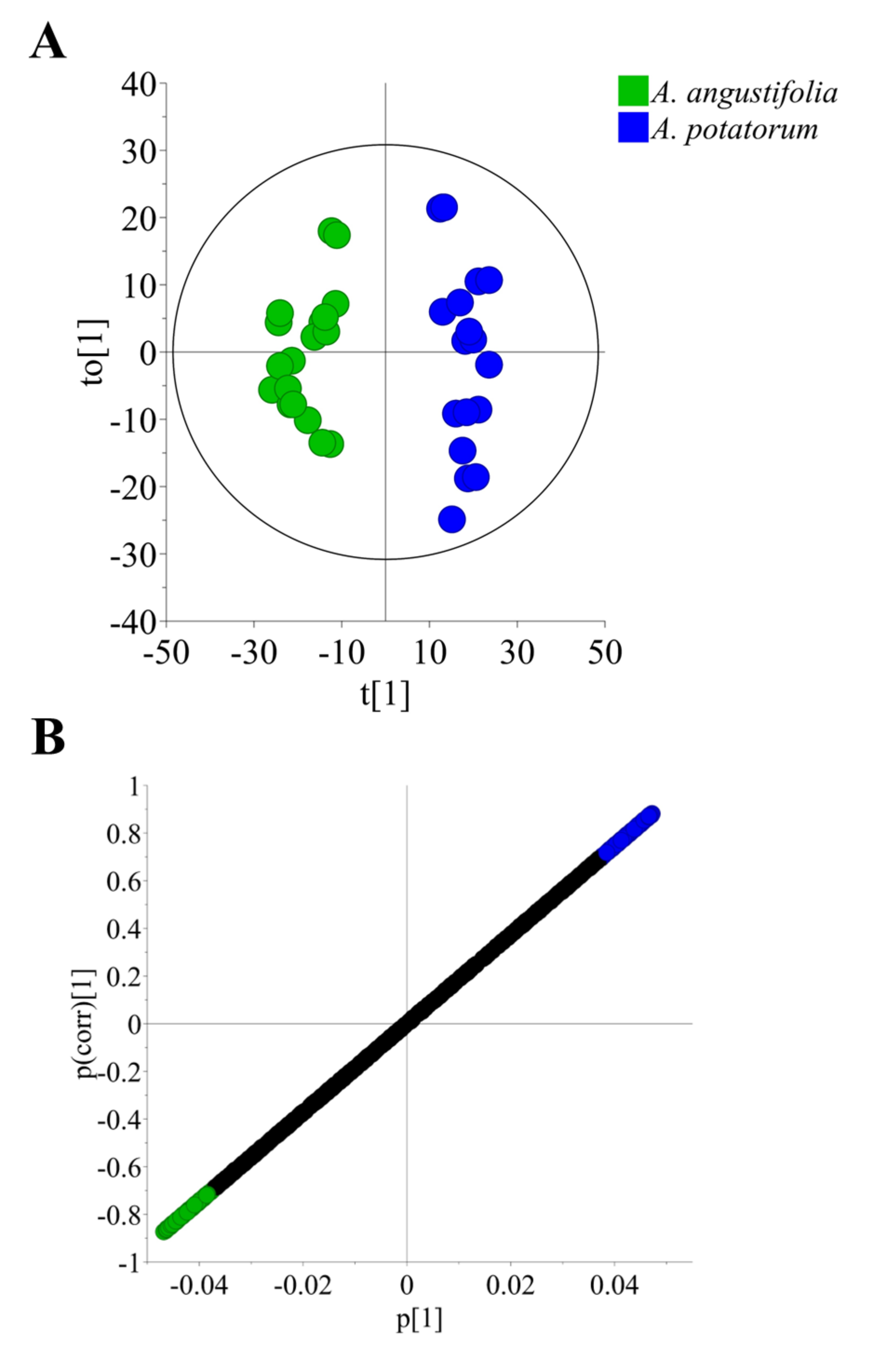

Supplementary figure S3. Supervised multivariate data analysis of fructans of *Agave potatorum* and *Agave angustifolia*. **(A)** Orthogonal projection to latent structures discriminant analysis (OPLS-DA) of complete fructan extracts (CF) obtained from *A. angustifolia* and *A. potatorum* specimens, the model used agave species as classes. **(B)** S-plot of the OPLS-DA model separated according to agave species. Green dots represent the most correlated variables to *A. angustifolia,* and the blue dots represent the most correlated variables to *A. potatorum*.

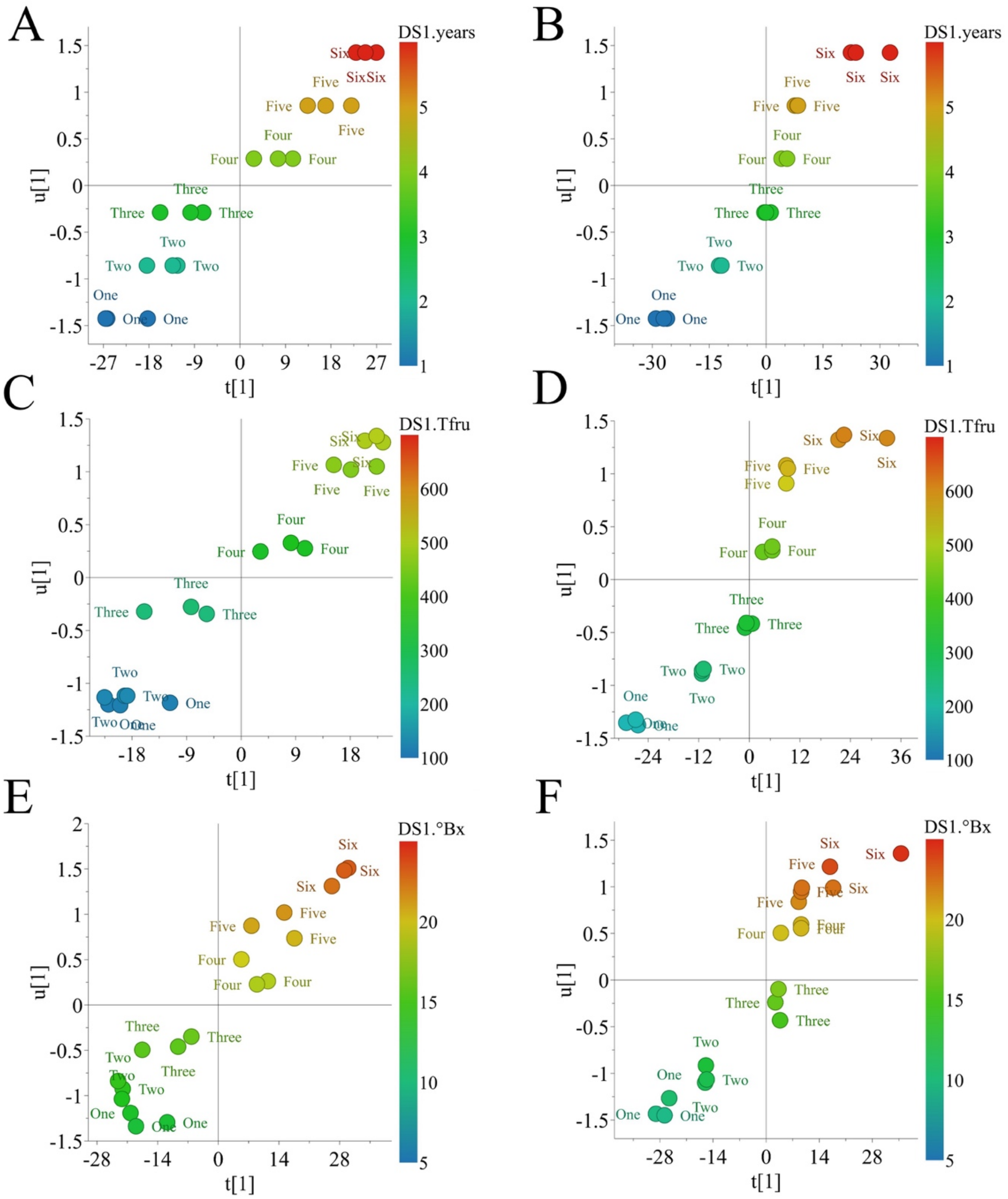

Supplementary figure S4. Correlation multivariate data analysis of fructans extracted from *Agave potatorum* and *Agave angustifolia*. **(A)** Orthogonal projection to latent structures (OPLS)-analysis of complete fructan extracts (CF) and age from *A. potatorum*. **(B)** OPLS analysis of aFOS and age from *A. angustifolia*. **(C)** OPLS analysis of CF and Tfru from *A. potatorum*. **(D)** OPLS analysis of aFOS and Tfru of *A. angustifolia*. **(E)** OPLS analysis of CF and ºBrix of *A. potatorum*. **(F)** OPLS analysis of aFOS and ºBrix of *A. angustifolia*.

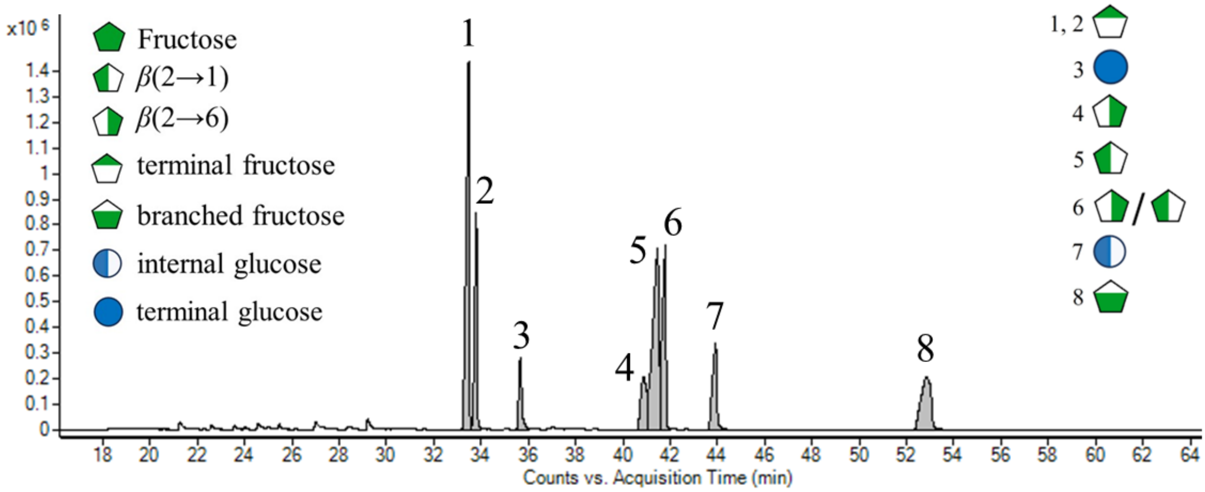

Supplementary figure S5. Typical chromatogram obtained after partially alditol acetate derivatization of agavins. The chromatogram belongs to a high polymerization degree fraction obtained from a six-year-old *A. potatorum* specimen, which confirm the presence of agavins in the extracts.

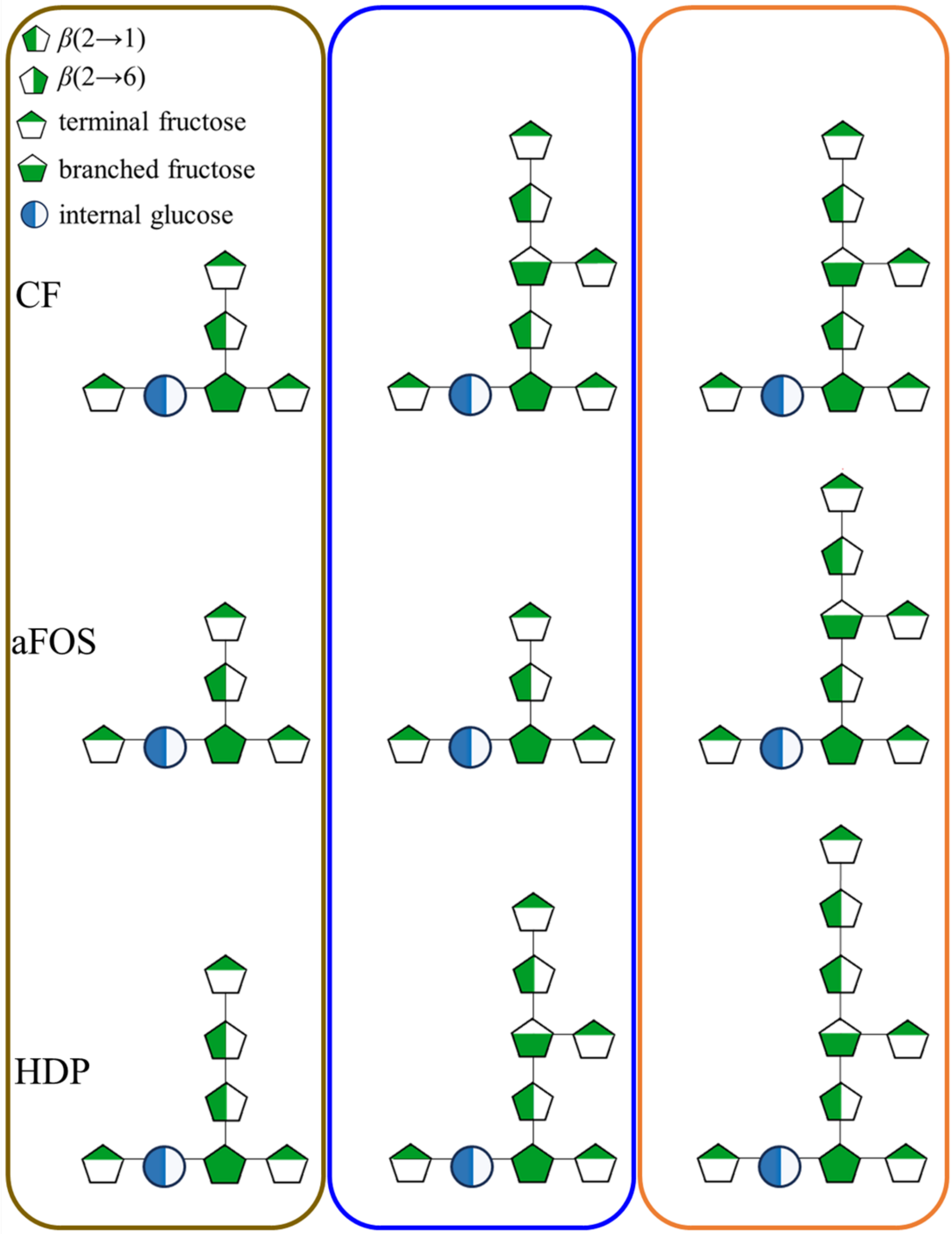

Supplementary figure S6. Predicted general agavin structures from partially methylated alditol acetate derivates of complete fructan extracts (CF), agavin fructooligosaccharides (aFOS) fractions and high polymerization degree (HDP)-fructans of one (brown box), three (blue box), and six-year-old (orange box) specimens of *Agave angustifolia*.

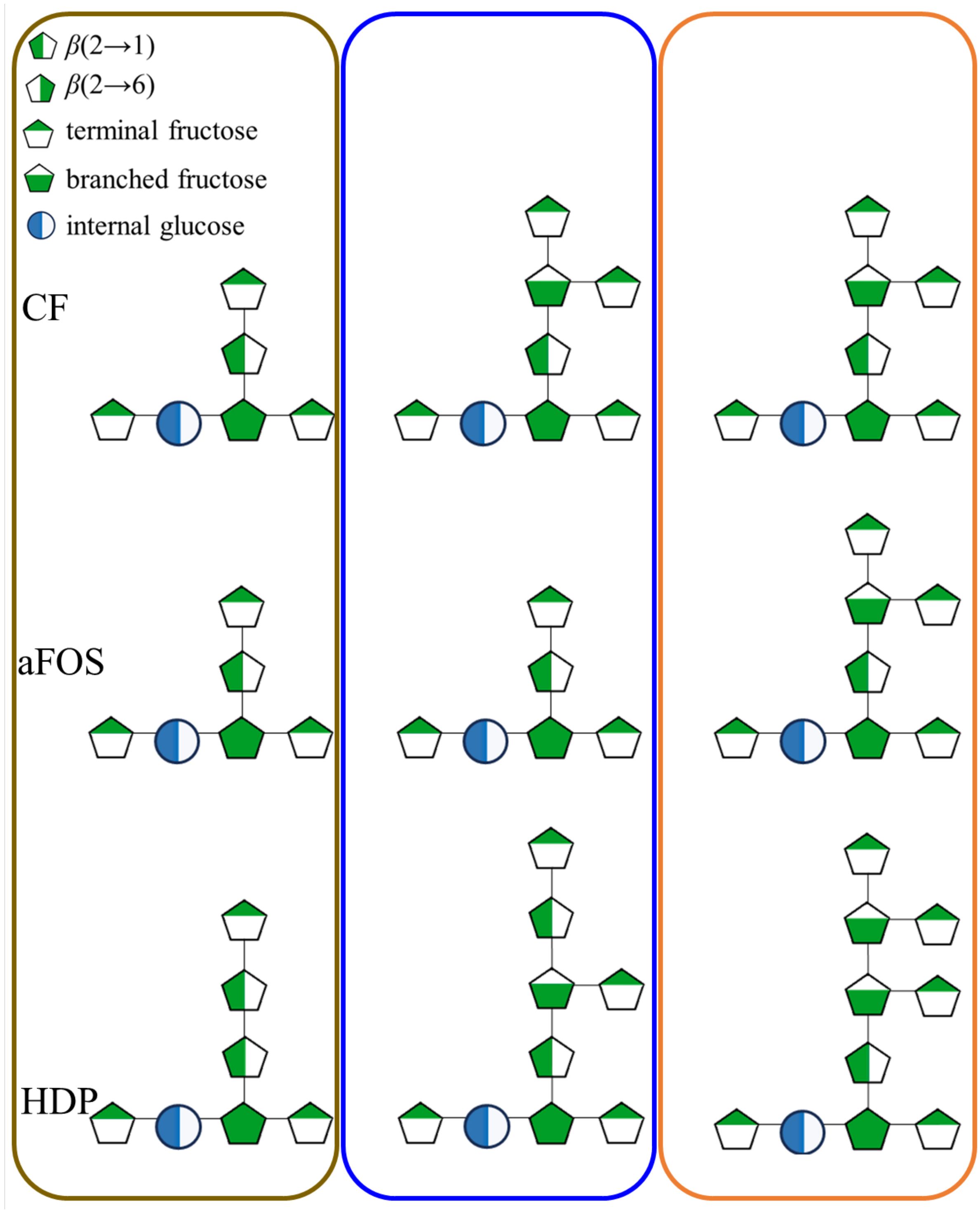

Supplementary figure S7. Predicted general agavin structures from partially methylated alditol acetate derivates of complete fructan (CF) extracts, agavin fructooligosaccharide (aFOS) fractions and high polymerization degree (HDP) fractions of one (brown box), three (blue box), and six-year-old (orange box) specimens of *Agave potatorum*.

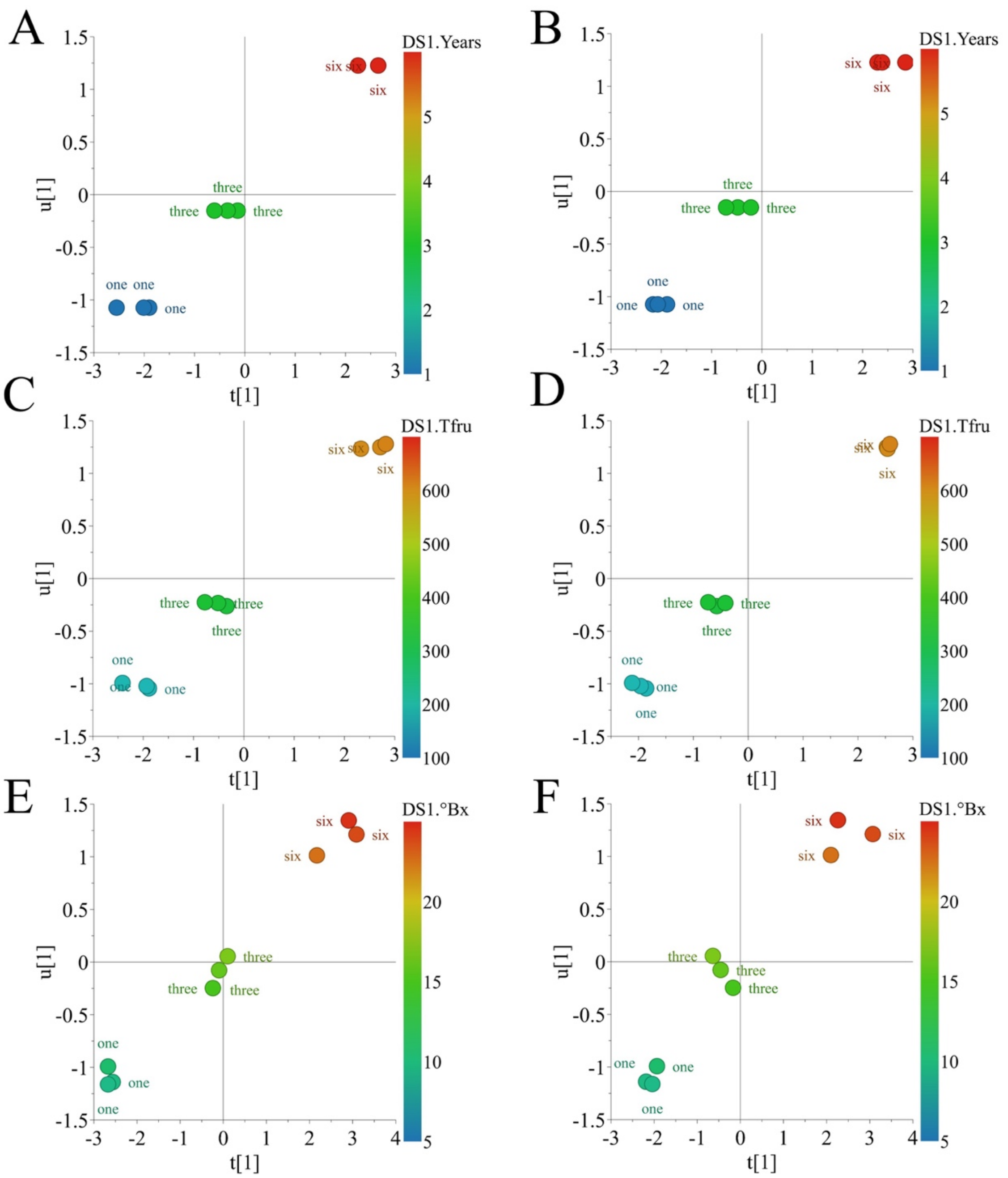

Supplementary figure 8. Correlation multivariate data analysis of fructans extracted from *Agave potatorum* and *Agave angustifolia* derivatized to partially methylated alditol acetates (PMAAs). **(A)** Orthogonal projection to latent structures (OPLS)-analysis of PMMA-derivatives of CF and age of *A. potatorum*. **(B)** OPLS analysis of PMAAs derivatives of aFOS and age of *A. angustifolia*. **(C)** OPLS analysis of of PMAAs derivatives of aFOS and Tfru of *A. potatorum*. **(D)** OPLS analysis of PMAAs derivatives of aFOS and Tfru of *A. angustifolia*. **(E)** OPLS analysis of PMAAs derivatives of CF and ºBrix of *A. potatorum*. **(F)** OPLS analysis of aFOS and ºBrix of *A. angustifolia*.

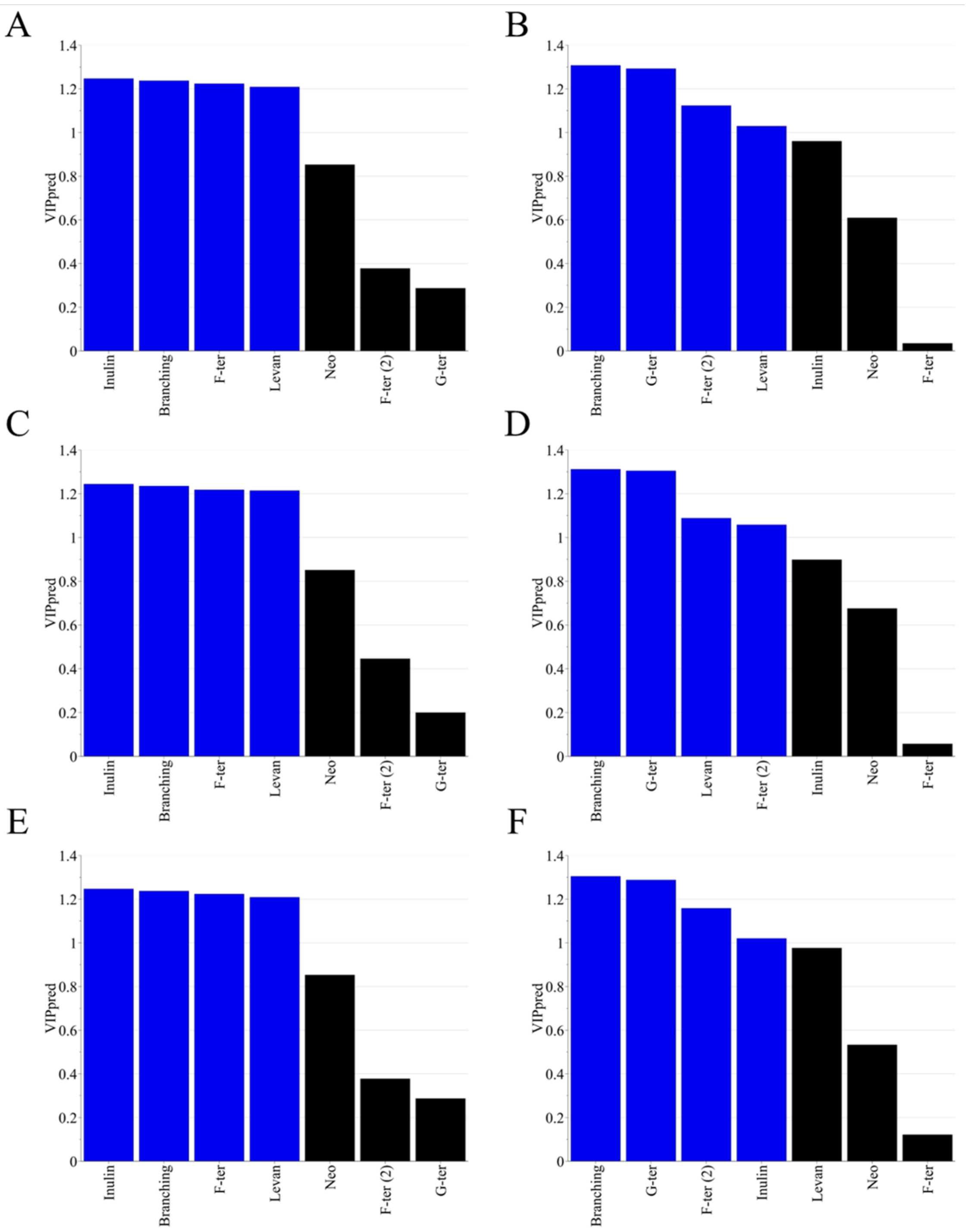

Supplementary figure S9. VIP*pred*-plot of an **(A)** OPLS analysis for the correlation between PMAA-derivatives data of CF and age of *Agave potatorum*. **(B)** OPLS analysis for the correlation between PMAA-derivatives data of aFOS and age of *Agave angustifolia*. **(C)** OPLS analysis for the correlation between PMAA-derivatives data of aFOS and Tfry of *A. potatorum*. **(D)** OPLS analysis for the correlation between PMAA-derivatives data of aFOS and Tfru of *A. angustifolia*. **(E)** OPLS analysis for the correlation between PMAA-derivatives of CF data and ºBrix of *A. potatorum*. **(F)** OPLS analysis for the correlation between PMAA-derivatives of aFOS data and ºBrix of *A. angustifolia*.

A

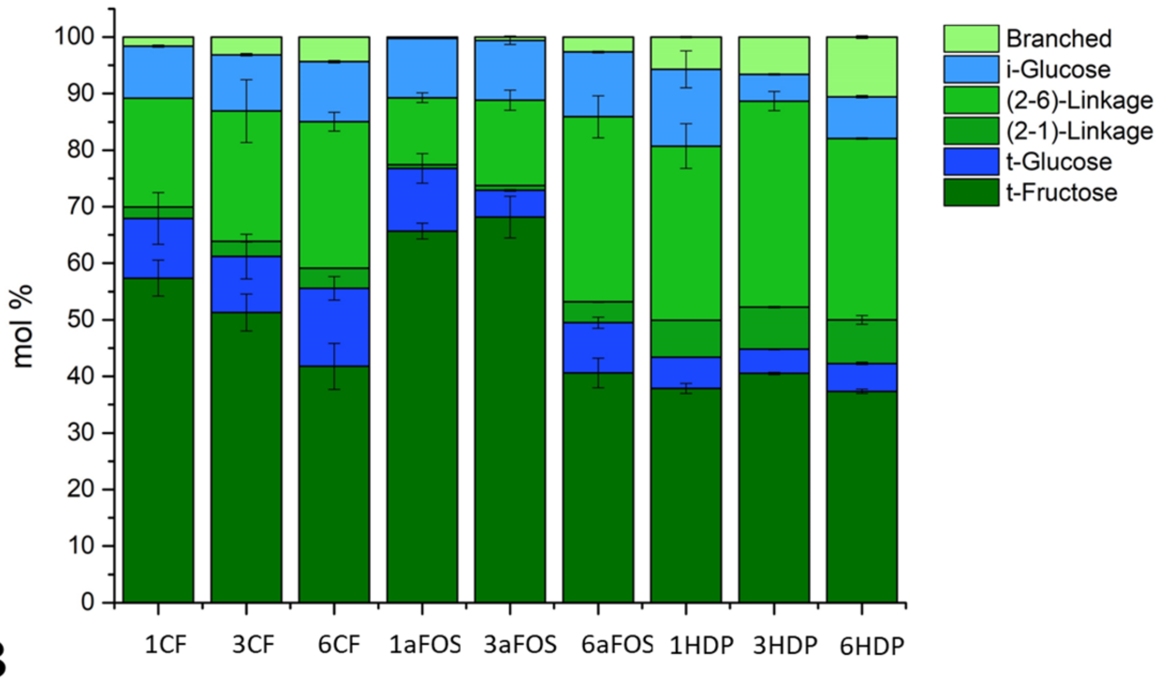

B

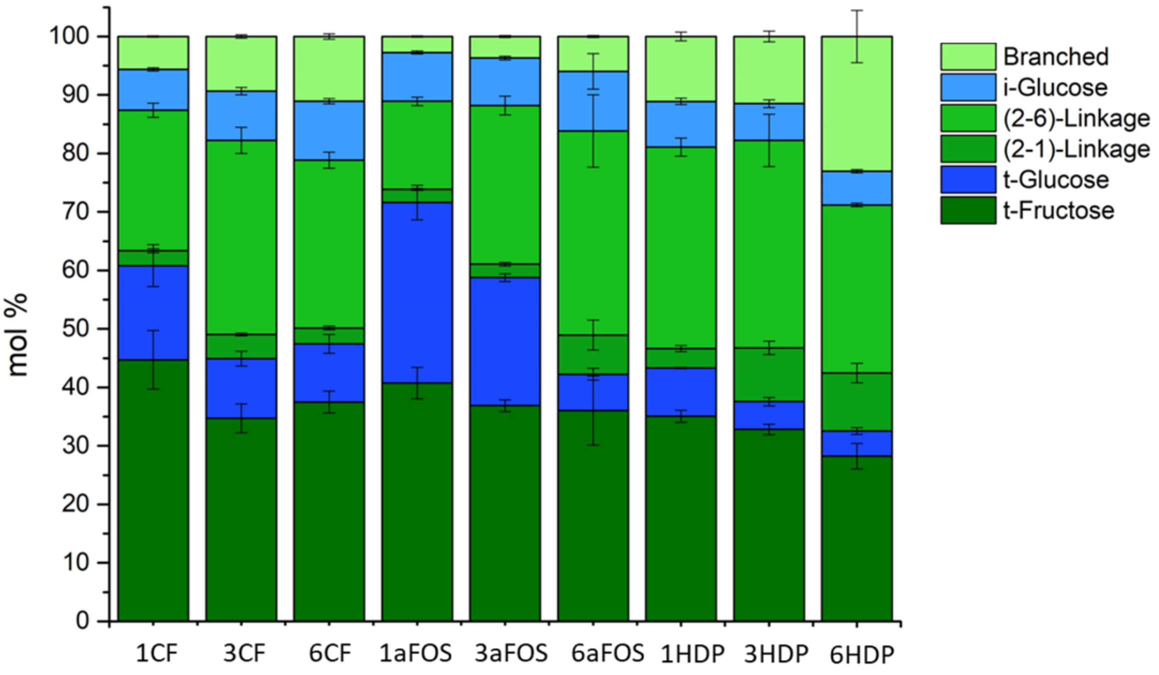

Supplementary figure S10. Molar contribution for each glycosidic linkage type present in the partially methylated alditol acetate (PMAA)-derivates chromatogram of **(A)** *Agave potatorum* and **(B)** *Agave angustifolia*. Branched, it indicates branching fructose; i-Glucose, it indicates internal glucose in agavins; (2-6)-Linkage, it indicates $\beta(2\rightarrow6)$ moieties in levan branches; (2-1)-Linkage, it indicates $\beta(2\rightarrow1)$ moieties in inulin chains; t-Glucose, it indicates terminal glucose present in graminan contained in agave specimens; t-Fructose, it indicates terminal fructose at the end of inulin or levan branches. CF means complete fructan extracts, aFOS means agavin fructooligosaccharide fraction, and HDP means high polymerization degree fructan fraction. Numbers 1, 3, and 6 indicate the specimens' age.

Supplementary table S1. Mass fragmentation data of glycosidic linkages present in *Agave potatorum* and *Agave angustifolia*.

Peak	Rt[a]	Derivative	Linkage type	Fragmentation Pattern (*m/z*)
1	33.5	2,5-di-O-acetyl-(2-deuterio)-1,3,4,6-tetra-O-methyl-D-mannitol	*t*-*β*-D-Fru*f*	129(100), 162(45.3), 161(30.1), 87(23.2), 101(13.3), 102(11.9), 75(10.1), 145(9.0), 72(7.2), 146(8.1)
2	33.8	2,5-di-O-acetyl-(2-deuterio)-1,3,4,6-tetra-O-methyl-D-glucitol	*t*-*β*-D-Fru*f*	129(100), 162(40.1), 161(35.2), 87(23.8), 101(13.8), 102(11.9), 75(10.2), 72(8.5), 146(6.5), 145(6.0)
3	35.7	1,5-di-O-acetyl-(1-deuterio)-2,3,4,6-tetra-O-thylglucitol	*t*-*α*-D-Glc*p*	102(100), 129(71.1), 145(55.0), 118(53.7), 101(51.4), 71(34.2), 87(35.8), 162(33.9), 161(31.2), 205(20.1), 72(1.65)
4	40.8	2,5,6-tri-O-acetyl-(2-deuterio)-1,3,4-tri-O-methylmannitol	(2→6)-*β*-D-Fru*f*	129(100), 162(41.0), 87(31.8), 189(16.2), 99(13.0), 102(11.7), 75(8.61), 72(6.30), 71(3.72), 60(1.99)
5	41.6	1,2,5-tri-O-acetyl-(2-deuterio)-3,4,6-tri-O-methylmannitol	(2→1)-*β*-D-Fru*f*	129 (100), 87 (29.8), 190 (23.1), 161 (22.1), 101 (10.9), 100 (10.2), 75 (6.85), 145 (6.60), 71 (6.38), 72 (5.21)
6	41.9	2,5,6-tri-O-acetyl-(2-deuterio)-1,3,4-tri-O-methylglucitol 1,2,5-tri-O-acetyl-(2-deuterio)-3,4,6-tri-O-methylglucitol	(2→6/1)-*β*-D-Fru*f*	129 (100), 87 (29.9), 161 (22.1), 190 (17.2), 162 (11.2), 101 (9.57), 100 (8.51), 189 (6.72), 71 (6.03), 75 (6.56), 72 (5.19), 118 (1.47)
7	44.1	1,5,6-tri-O-acetyl-(1-deuterio)-2,3,4-tri-O-methylglucitol	*i*-*α*-D-Glc*p*	102 (100), 118 (70.1), 129 (67.6), 87 (48.2), 101 (28.1), 162 (25.8), 189 (19.8), 71 (14.2), 233 (7.84), 145 (0.83)
8	52.9	1,2,5,6-tetra-O-acetyl-(2-deuterio)-3,4-di-O-methylhexitol	1,6-di-*β*-D-Fru*f*	129 (100), 87 (37.1), 190 (23.5), 189 (16.0), 99 (11.0), 100 (10.8), 72 (4.20), 71 (3.17), 60 (1.21)

[a]Rt, retention time expressed in minutes.

Supplementary table S2. Top 50 correlated variables determined by the predictive variable importance for the projection (VIP_{pred})-plot of the orthogonal projection to latent structures model for the correlation between agave age and mid infrared pattern variation in *Agave angustifolia*.

Wave number (cm^{-1})	VIP_{pred} score
1060.19	1.62127
1059.71	1.62103
1060.67	1.61388
1059.23	1.61025
1061.16	1.60455
992.212	1.60325
991.73	1.60309
1063.08	1.60053
1009.09	1.60038
1063.57	1.59967
1062.6	1.59836
1061.64	1.59836
1062.12	1.59681
992.694	1.592
1058.74	1.58919
1064.05	1.58858
1205.31	1.58799
991.248	1.58685
1077.55	1.57918
1008.6	1.57833
1205.79	1.57724
1078.03	1.57505
993.176	1.5743
1077.07	1.57344
1204.83	1.57263
1237.13	1.56852
1236.65	1.56025
1058.26	1.55906
1221.22	1.55902
1076.58	1.55883
1078.51	1.55874
1064.53	1.55787
1221.7	1.55646
993.658	1.55478
990.765	1.55182
1009.57	1.55035

**1237.61**	1.5503
**1236.17**	1.54237
**1204.35**	1.54001
**994.14**	1.53637
**1076.1**	1.53532
**1220.74**	1.53276
**934.357**	1.53072
**1206.28**	1.52953
**1078.99**	1.52672
**1235.69**	1.52281
**1057.78**	1.51991
**1222.19**	1.51926
**994.622**	1.51885
**934.839**	1.51583

Supplementary table S3. Top 50 correlated variables determined by the predictive variable importance for the projection (VIP_{pred})-plot of the orthogonal projection to latent structures model for the correlation between agave total fructan content and mid infrared pattern variation in *Agave angustifolia*.

Wave number (cm^{-1})	VIP_{pred} score
**1059.71**	1.63294
**1060.19**	1.63151
**1059.23**	1.62283
**1060.67**	1.62214
**1205.31**	1.61223
**1061.16**	1.61126
**992.212**	1.60896
**1063.08**	1.60819
**991.73**	1.60713
**1077.55**	1.60699
**1063.57**	1.60666
**1009.09**	1.60666
**1062.6**	1.6056
**1061.64**	1.60446
**1077.07**	1.60343
**1062.12**	1.60326
**1058.74**	1.60102
**1204.83**	1.60045
**1078.03**	1.60037
**992.694**	1.59844
**1205.79**	1.59778
**1064.05**	1.59341
**1076.58**	1.59067
**991.248**	1.58835
**1078.51**	1.58135
**1221.22**	1.58076
**993.176**	1.58072
**1008.6**	1.57931
**1221.7**	1.57833
**1204.35**	1.57144
**1237.13**	1.57051
**1076.1**	1.56896
**1058.26**	1.56867
**1236.65**	1.56286
**1009.57**	1.56196
**993.658**	1.56085

**1064.53**	1.55913
**1220.74**	1.55284
**990.765**	1.55023
**1237.61**	1.55005
**1078.99**	1.54652
**1206.28**	1.54616
**1236.17**	1.54416
**994.14**	1.54225
**1222.19**	1.53927
**1075.62**	1.53754
**934.357**	1.53319
**1203.86**	1.52869
**933.875**	1.52669
**1057.78**	1.52602

Supplementary table S4. Top 50 correlated variables determined by the predictive variable importance for the projection (VIP_{pred})-plot of the orthogonal projection to latent structures model for the correlation between agave brix degrees and mid infrared pattern variation in *Agave angustifolia*.

Wave number (cm^{-1})	VIP_{pred} score
**1059.71**	1.5839
**1063.57**	1.58325
**1063.08**	1.58317
**1060.19**	1.58257
**1009.57**	1.57874
**1062.6**	1.57725
**992.694**	1.57616
**1059.23**	1.57492
**1060.67**	1.57489
**1009.09**	1.57478
**1078.51**	1.57222
**992.212**	1.57203
**1064.05**	1.57116
**993.176**	1.57033
**1062.12**	1.56996
**1061.16**	1.56746
**1078.99**	1.56642
**1061.64**	1.56559
**1078.03**	1.56363
**993.658**	1.56153
**1058.74**	1.55519
**994.14**	1.55499
**994.622**	1.55175
**991.73**	1.55175
**995.105**	1.54804
**1077.55**	1.54403
**1079.48**	1.54191
**1064.53**	1.53983
**995.587**	1.53807
**933.875**	1.53777
**934.357**	1.52891
**1058.26**	1.52578
**1204.83**	1.52508
**996.069**	1.51789
**1205.31**	1.51582
**1077.07**	1.51566

**1204.35**	1.51402
**991.248**	1.51139
**933.393**	1.51074
**1010.05**	1.50737
**1008.6**	1.50572
**1124.31**	1.49755
**1123.83**	1.49705
**1143.6**	1.49642
**1124.8**	1.49555
**1236.65**	1.49432
**1123.35**	1.49408
**1079.96**	1.49356
**934.839**	1.49317
**1125.28**	1.49156

Supplementary table S5. Top 50 correlated variables determined by the predictive variable importance for the projection (VIP_{pred})-plot of the orthogonal projection to latent structures model for the correlation between agave age and mid infrared pattern variation in *Agave potatorum*.

Wave number (cm^{-1})	VIP_{pred} score
1175.42	1.45004
1175.9	1.4492
1092.98	1.44797
1092.49	1.44558
1093.46	1.44435
1174.94	1.44088
1176.38	1.43898
1092.01	1.43755
1093.94	1.43408
1091.53	1.42457
1174.45	1.42312
1176.86	1.42199
955.088	1.41985
1024.51	1.41719
1094.42	1.41662
955.57	1.4161
1024.03	1.41482
954.606	1.4099
1091.05	1.40762
1025	1.4061
1177.35	1.40209
956.052	1.40092
1173.97	1.39939
1129.62	1.39924
978.23	1.39739
1130.1	1.39708
1129.14	1.3964
978.712	1.39637
1023.55	1.3956
977.748	1.395
1094.9	1.39335
979.195	1.39291
1128.65	1.39093
1090.56	1.3877
977.266	1.38764
979.677	1.38728

**954.124**	1.38684
**1130.58**	1.38629
**1025.48**	1.3838
**1128.17**	1.38359
**1177.83**	1.38315
**998.479**	1.38018
**998.961**	1.37952
**980.159**	1.37923
**956.535**	1.3767
**1127.69**	1.37384
**976.784**	1.37301
**1173.49**	1.37191
**999.444**	1.36961
**1095.39**	1.36935

Supplementary table S6. Top 50 correlated variables determined by the predictive variable importance for the projection (VIP_{pred})-plot of the orthogonal projection to latent structures model for the correlation between agave total fructan content and mid infrared pattern variation in *Agave potatorum*.

Wave number (cm^{-1})	VIP_{pred} score
**1174.45**	1.54604
**1173.97**	1.54255
**1174.94**	1.54158
**1173.49**	1.53112
**1175.42**	1.52846
**1173.01**	1.50941
**1175.9**	1.50679
**1176.38**	1.47809
**1172.53**	1.46939
**955.088**	1.46925
**1093.46**	1.46637
**1092.98**	1.46607
**954.606**	1.46345
**976.784**	1.46168
**977.266**	1.4613
**955.57**	1.45862
**1093.94**	1.45724
**1092.49**	1.45724
**977.748**	1.4527
**976.302**	1.45091
**997.997**	1.44603
**1176.86**	1.44532
**997.515**	1.44407
**954.124**	1.4414
**1092.01**	1.44062
**978.23**	1.4385
**1094.42**	1.43823
**956.052**	1.43443
**1129.14**	1.43066
**1129.62**	1.42837
**998.479**	1.42805
**975.82**	1.42558
**1128.65**	1.4255
**978.712**	1.42091
**946.41**	1.41976
**946.892**	1.41894

**997.033**	1.41794
**1091.53**	1.41727
**1130.1**	1.41634
**1128.17**	1.41405
**1177.35**	1.41221
**1094.9**	1.41149
**953.642**	1.40879
**979.195**	1.40166
**956.535**	1.39959
**998.961**	1.39777
**1127.69**	1.39645
**1172.04**	1.3925
**1130.58**	1.39161
**1091.05**	1.38852

Supplementary table S7. Top 50 correlated variables determined by the predictive variable importance for the projection (VIP_{pred})-plot of the orthogonal projection to latent structures model for the correlation between agave brix degrees and mid infrared pattern variation in *Agave potatorum*.

Wave number (cm^{-1})	VIP_{pred} score
**1175.42**	1.46141
**1174.94**	1.45973
**1175.9**	1.45359
**977.266**	1.45325
**976.784**	1.45099
**1174.45**	1.44837
**977.748**	1.44791
**976.302**	1.43927
**1176.38**	1.43772
**978.23**	1.43665
**955.088**	1.43612
**955.57**	1.43342
**997.997**	1.42963
**997.515**	1.429
**1173.97**	1.42765
**954.606**	1.42566
**978.712**	1.42111
**956.052**	1.41987
**1176.86**	1.41646
**975.82**	1.41571
**998.479**	1.40771
**1129.14**	1.40642
**1129.62**	1.40462
**1128.65**	1.40369
**979.195**	1.40277
**954.124**	1.40252
**997.033**	1.40168
**1011.02**	1.4012
**956.535**	1.39778
**1173.49**	1.39709
**1128.17**	1.39689
**1130.1**	1.39648
**1177.35**	1.39308
**1011.5**	1.38972
**1127.69**	1.38526
**979.677**	1.38273

**1130.58**	1.37917
**975.338**	1.3769
**953.642**	1.3721
**998.961**	1.37165
**1177.83**	1.37054
**1010.53**	1.36876
**957.017**	1.36823
**1127.21**	1.36771
**1011.98**	1.36566
**1217.36**	1.36306
**980.159**	1.36163
**1060.67**	1.35977
**1092.49**	1.35912
**1092.98**	1.35805

Supplementary table S8. Top 50 correlated variables determined by the predictive variable importance for the projection (VIP_{pred})-plot of the orthogonal projection to latent structures model for the correlation between inulin moieties degree determined by gas chromatography-mass spectrometry and mid infrared pattern variation in *Agave potatorum*.

Wave number (cm^{-1})	VIP_{pred} score
**1077.55**	1.24139
**1026.44**	1.24085
**1026.93**	1.2405
**955.57**	1.24014
**1038.5**	1.24004
**1025.96**	1.23978
**1038.98**	1.23924
**1078.03**	1.2389
**1038.01**	1.23886
**1027.41**	1.23868
**1039.46**	1.23803
**1025.48**	1.23789
**956.052**	1.23749
**955.088**	1.23706
**1093.94**	1.23704
**1094.42**	1.23698
**1106.96**	1.23681
**1106.47**	1.23672
**1093.46**	1.23647
**1105.99**	1.23631
**1107.44**	1.23626
**1077.07**	1.23618
**1027.89**	1.23612
**1039.94**	1.23603
**1025**	1.23587
**1094.9**	1.23572
**1092.98**	1.23563
**1105.51**	1.2353
**1092.49**	1.23474
**1024.51**	1.23414
**1028.37**	1.2339
**1092.01**	1.2338
**1105.03**	1.23345
**1107.92**	1.23276
**1091.53**	1.23272
**1024.03**	1.23271

**919.893**	1.23264
**949.785**	1.23253
**995.105**	1.23247
**1028.85**	1.23241
**1095.39**	1.23237
**1037.53**	1.2322
**919.411**	1.23194
**1078.51**	1.23193
**995.587**	1.23175
**949.303**	1.23169
**920.375**	1.23153
**1091.05**	1.23143
**954.606**	1.23128
**1023.55**	1.23106

Supplementary table S9. Top 50 correlated variables determined by the predictive variable importance for the projection (VIP_{pred})-plot of the orthogonal projection to latent structures model for the correlation between levan moieties degree determined by gas chromatography-mass spectrometry and mid infrared pattern variation in *Agave potatorum*.

Wave number (cm^{-1})	VIP_{pred} score
**970.034**	1.24737
**970.516**	1.24703
**969.552**	1.24692
**1090.56**	1.2467
**1090.08**	1.24659
**1091.05**	1.24649
**1089.6**	1.24632
**1089.12**	1.24598
**1091.53**	1.24597
**970.998**	1.24584
**969.07**	1.24561
**1088.64**	1.24548
**1092.01**	1.24528
**1092.49**	1.24462
**1088.16**	1.24439
**1092.98**	1.24416
**1093.46**	1.24392
**1093.94**	1.2438
**1094.42**	1.24359
**955.088**	1.24337
**971.481**	1.24334
**968.588**	1.24323
**1094.9**	1.243
**949.303**	1.24235
**948.821**	1.2421
**954.606**	1.24208
**995.587**	1.24187
**1087.67**	1.24168
**955.57**	1.24163
**996.069**	1.24162
**1095.39**	1.24157
**949.785**	1.24124
**948.339**	1.24093
**1106.47**	1.23994
**1106.96**	1.23987
**968.106**	1.23923

**978.712**	1.2392
**996.551**	1.23918
**1105.99**	1.23897
**1027.41**	1.23885
**954.124**	1.23884
**1107.44**	1.23874
**1027.89**	1.2387
**947.856**	1.23849
**950.267**	1.23847
**995.105**	1.23843
**1095.87**	1.23838
**971.963**	1.23805
**1022.59**	1.23775
**1026.93**	1.23771

Supplementary table S10. Top 50 correlated variables determined by the predictive variable importance for the projection (VIP_{pred})-plot of the orthogonal projection to latent structures model for the correlation between branching fructose degree determined by gas chromatography-mass spectrometry and mid infrared pattern variation in *Agave potatorum*.

Wave number (cm^{-1})	VIP_{pred} score
**1093.94**	1.2287
**1094.42**	1.22867
**1038.5**	1.22844
**1026.93**	1.22835
**1093.46**	1.22825
**1038.98**	1.22823
**1039.46**	1.22822
**1026.44**	1.22807
**1039.94**	1.22802
**1105.03**	1.22802
**1105.51**	1.22793
**1094.9**	1.22779
**1092.98**	1.22745
**1027.41**	1.22741
**1038.01**	1.22716
**1025.96**	1.22672
**1104.55**	1.22664
**1078.03**	1.22638
**1092.49**	1.22632
**1105.99**	1.2263
**1027.89**	1.2259
**1077.55**	1.22557
**1095.39**	1.22538
**1025.48**	1.22499
**1040.42**	1.22487
**1028.37**	1.22486
**1092.01**	1.22478
**1028.85**	1.22465
**1023.55**	1.22447
**1169.15**	1.22424
**949.785**	1.22421
**1104.06**	1.22417
**1029.33**	1.22394
**1168.67**	1.22392
**1169.63**	1.22379
**950.267**	1.22378

**1024.03**	1.22373
**1025**	1.22366
**1023.07**	1.22348
**955.088**	1.22342
**1106.47**	1.22331
**1024.51**	1.22323
**1091.53**	1.22273
**1078.51**	1.22268
**949.303**	1.22267
**1168.19**	1.22259
**1170.12**	1.22256
**907.358**	1.22255
**954.606**	1.22238
**950.749**	1.2213

Supplementary table S11. Top 50 correlated variables determined by the predictive variable importance for the projection (VIP_{pred})-plot of the orthogonal projection to latent structures model for the correlation between terminal glucose degree determined by gas chromatography-mass spectrometry and mid infrared pattern variation in *Agave angustifolia*.

Wave number (cm^{-1})	VIP_{pred} score
**1044.28**	1.48982
**1043.8**	1.48817
**1044.76**	1.48811
**1045.24**	1.48468
**1043.32**	1.48029
**1045.73**	1.47964
**d1065.49**	1.47837
**1038.01**	1.47763
**1237.61**	1.47723
**1038.5**	1.47653
**1065.01**	1.47537
**1075.14**	1.47499
**1074.66**	1.47454
**1238.09**	1.47337
**1059.23**	1.47328
**1059.71**	1.47319
**1046.21**	1.472
**1008.6**	1.47168
**1065.98**	1.47101
**1058.74**	1.46911
**1205.79**	1.46883
**1060.19**	1.46735
**1074.17**	1.46427
**1042.83**	1.46399
**1075.62**	1.4633
**1168.19**	1.46163
**1058.26**	1.4615
**1167.7**	1.4614
**1008.12**	1.46048
**1046.69**	1.46048
**1064.53**	1.4604
**1066.46**	1.45973
**1237.13**	1.45922
**1038.98**	1.45903
**1168.67**	1.45856
**1206.28**	1.45812

**1233.76**	1.458
**1037.53**	1.45792
**1234.24**	1.45641
**1060.67**	1.4557
**1167.22**	1.45441
**1169.15**	1.45328
**991.248**	1.45295
**1142.64**	1.45286
**1066.94**	1.45138
**1205.31**	1.45045
**991.73**	1.4492
**1057.78**	1.44888
**935.803**	1.44865
**1121.42**	1.44861

Supplementary table S12. Top 50 correlated variables determined by the predictive variable importance for the projection (VIP_{pred})-plot of the orthogonal projection to latent structures model for the correlation between branching fructose degree determined by gas chromatography-mass spectrometry and mid infrared pattern variation in *Agave angustifolia*.

Wave number (cm^{-1})	VIP_{pred} score
**1238.09**	1.48827
**1065.98**	1.48262
**1065.49**	1.48152
**1237.61**	1.48051
**1044.76**	1.47926
**1045.24**	1.4787
**1044.28**	1.47729
**1066.46**	1.47695
**1045.73**	1.47592
**1038.01**	1.47325
**1043.8**	1.47119
**1066.94**	1.4702
**1046.21**	1.47004
**1065.01**	1.46856
**1142.64**	1.46788
**1038.5**	1.4667
**1008.12**	1.46524
**1205.79**	1.46295
**1008.6**	1.4622
**1238.58**	1.46122
**1046.69**	1.45982
**1075.14**	1.45912
**1043.32**	1.4584
**1206.28**	1.45823
**1037.53**	1.45755
**1067.42**	1.45704
**1142.15**	1.45698
**1074.66**	1.45376
**1237.13**	1.45343
**1059.23**	1.4523
**1075.62**	1.45135
**1059.71**	1.45078
**1058.74**	1.44984
**1007.64**	1.44558
**1233.76**	1.44507
**1047.17**	1.44436

**1222.67**	1.44409
**1058.26**	1.44403
**1038.98**	1.44376
**1060.19**	1.44368
**1234.24**	1.44357
**1064.53**	1.44355
**1224.6**	1.44203
**1223.15**	1.44191
**1222.19**	1.44094
**1143.12**	1.44063
**1224.11**	1.44018
**1223.63**	1.43939
**1205.31**	1.43911
**1074.17**	1.43817
